# Harmonization of brain PET images in multi-center PET studies using Hoffman phantom scan

**DOI:** 10.1186/s40658-023-00588-x

**Published:** 2023-10-31

**Authors:** Mahnaz Shekari, Eline E. Verwer, Maqsood Yaqub, Marcel Daamen, Christopher Buckley, Giovanni B. Frisoni, Pieter Jelle Visser, Gill Farrar, Frederik Barkhof, Juan Domingo Gispert, Ronald Boellaard, the AMYPAD Consortium

**Affiliations:** 1 Barcelonaβeta Brain Research Center (BBRC), Pasqual Maragall Foundation, Barcelona, Spain; 2 IMIM (Hospital del Mar Medical Research Institute), Barcelona, Spain; 3 Universitat Pompeu Fabra, Barcelona, Spain; 4 Department of Radiology and Nuclear Medicine, Amsterdam, University Medical Centers, Location VUmc, De Boelelaan 1117, 1081 HV Amsterdam, The Netherlands; 5 German Center for Neurodegenerative Diseases (DZNE), Bonn, Germany; 6 GE Healthcare, Amersham, UK; 7 Laboratory of Neuroimaging of Aging (LANVIE), University of Geneva, Geneva, Switzerland; 8 Memory Center, Department of Rehabilitation and Geriatrics, University Hospitals and University of Geneva, Geneva, Switzerland; 9 Alzheimer Center Amsterdam, Neurology, Vrije Universiteit Amsterdam, Amsterdam UMC, Location VUmc, Amsterdam, The Netherlands; 10 Amsterdam Neuroscience, Neurodegeneration, Amsterdam, The Netherlands; 11 Alzheimer Center Limburg, School for Mental Health and Neuroscience, Maastricht University, Maastricht, The Netherlands; 12 Department of Neurobiology, Care Sciences and Society, Division of Neurogeriatrics, Karolinska Institutet, Stockholm, Sweden; 13 Queen Square Institute of Neurology, University College London, London, UK; 14 Centro de Investigación Biomédica en Red Bioingeniería, Biomateriales y Nanomedicina, (CIBER-BBN), Barcelona, Spain

**Keywords:** Brain PET, Harmonization, Neuroimaging, Quantification

## Abstract

**Background:**

Image harmonization has been proposed to minimize heterogeneity in brain PET scans acquired in multi-center studies. However, standard validated methods and software tools are lacking. Here, we assessed the performance of a framework for the harmonization of brain PET scans in a multi-center European clinical trial.

**Method:**

Hoffman 3D brain phantoms were acquired in 28 PET systems and reconstructed using site-specific settings. Full Width at Half Maximum (FWHM) of the Effective Image Resolution (EIR) and harmonization kernels were estimated for each scan. The target EIR was selected as the coarsest EIR in the imaging network. Using “Hoffman 3D brain Analysis tool,” indicators of image quality were calculated before and after the harmonization: The Coefficient of Variance (COV%), Gray Matter Recovery Coefficient (GMRC), Contrast, Cold-Spot RC, and left-to-right GMRC ratio. A COV% ≤ 15% and Contrast ≥ 2.2 were set as acceptance criteria. The procedure was repeated to achieve a 6-mm target EIR in a subset of scans. The method’s robustness against typical dose-calibrator-based errors was assessed.

**Results:**

The EIR across systems ranged from 3.3 to 8.1 mm, and an EIR of 8 mm was selected as the target resolution. After harmonization, all scans met acceptable image quality criteria, while only 13 (39.4%) did before. The harmonization procedure resulted in lower inter-system variability indicators: Mean ± SD COV% (from 16.97 ± 6.03 to 7.86 ± 1.47%), GMRC Inter-Quartile Range (0.040–0.012), and Contrast SD (0.14–0.05). Similar results were obtained with a 6-mm FWHM target EIR. Errors of ± 10% in the DRO activity resulted in differences below 1 mm in the estimated EIR.

**Conclusion:**

Harmonizing the EIR of brain PET scans significantly reduced image quality variability while minimally affecting quantitative accuracy. This method can be used prospectively for harmonizing scans to target sharper resolutions and is robust against dose-calibrator errors. Comparable image quality is attainable in brain PET multi-center studies while maintaining quantitative accuracy.

**Supplementary Information:**

The online version contains supplementary material available at 10.1186/s40658-023-00588-x.

## Introduction

Quantification of brain positron emission tomography (PET) imaging is used as a biomarker in many clinical and research applications [1–9]. For instance, in clinical trials of anti-amyloid disease-modifying drugs for Alzheimer’s disease, semi-quantitative amyloid PET is used to assess treatment response [10], select participants for prevention trials, and guide dosing and treatment cessation schemes [11–13]. Standard uptake value (SUV) and standard uptake value ratio (SUVr) are the most common semi-quantitative metrics used for brain PET. These quantitative metrics are affected by many factors including reconstruction parameters, system specifications, and injected dose, among others as discussed in detail elsewhere [14]. In research multi-center studies, these factors introduce an additional source of variability, adding to the heterogeneity of the data and reducing the statistical power of PET quantitative endpoints. Therefore, it is necessary to develop methodologies and criteria to establish precision metrics that can render comparable quantitative brain PET outcomes across different imaging sites.

Several recommendations for acquiring data and reducing inter-system variability have been proposed for quantitative multi-center brain PET [15–18]. They commonly involve the optimization of reconstruction parameters with respect to a target quantitative image quality indicator, such as a target image resolution (mm Full Width at Half Maximum; FWHM), the recovery coefficient (RC), and the signal-to-noise ratio. To calculate these metrics, phantom scans with known geometries and activity distributions are typically acquired and compared to digital reference objects (DRO) [17, 19]. Using this approach, Ikari et al. conducted a study on 22 PET systems and they showed that achieving a contrast recovery higher than 55% and a COV% below 15% should be considered as acceptance criteria for brain PET image quality. Using these criteria is useful to prevent selecting poor reconstruction settings, and as a result, it improves quantitative accuracy and narrows down variabilities of quantitative and image quality indicators in multicenter studies. However, this procedure is intended to provide acceptable quality criteria, rather than performing a harmonization of the scans.

Despite all the efforts and initiatives, there are no universally accepted standard guidelines for harmonizing brain PET images in multi-center studies that encompass phantom selection, data acquisition, post-processing steps, and harmonization contrasts with the situation in oncological whole-body PET where harmonization standards and accreditation programs exist for accurate and reproducible PET outcomes [20–22]. Harmonization of oncological PET images is done using either NEMA NU2 anthropomorphic or Deluxe Jaszczak phantoms, with fillable spherical inserts representative of elevated focal uptakes. Several guidelines like EANM Research Ltd. (EARL) and the Japanese Society of Nuclear Medicine (JSNM) are available, describing the methodological considerations for harmonizing quantitative metrics across different imaging sites [20–22]. One of the key components of a standard guideline for the harmonization of quantitative PET outcomes is a procedure which is straightforward to implement. For instance, harmonization of oncological PET images is done using phantoms, where phantom preparation is a simple procedure. Moreover, quantitative metrics such as SUV, RC, and image quality indicators, such as COV% can be extracted from reconstructed phantom images either manually, by drawing a spherical volume of interest (VOI) using an image viewer software or with automatic programming scripts. This contrasts with the brain image harmonization, where extracting the relevant image quality indicators and quantitative metrics from the Hoffman brain phantom involves a significant amount of image processing with the lack of validated reference methods or software tools.

To overcome such limitations, here we present the results of using a software tool and quality criteria that have been recently developed to achieve comparable image quality and harmonize PET system performance [18]. Building on top of this software tool, in this study, we have additionally developed and evaluated the performance of a standardized framework for harmonizing brain PET scans in a current multi-center trial, Amyloid Imaging to Prevent Alzheimer's Disease (AMYPAD) involving 24 imaging centers with a total of 28 PET systems [23, 24]. A standard operational procedure (SOP) has been developed which covers the preparation of the 3D Hoffman brain phantom, scanning, analysis, and acceptance criteria. The procedure was designed to allow both prospective and retrospective harmonization and assessed the harmonization quality metric that best represented the global quality of the brain PET scans.

## Methods

### AMYPAD imaging network

The AMYPAD study aims to determine the value of amyloid PET imaging as a prognostic and diagnostic tool for Alzheimer’s disease. AMYPAD included two multi-center clinical trials, the Diagnostic and Patient Management Study and the Prognostic and Natural History Study [23, 25]. Both studies used ^18^F-Florbetaben (Neuraceq®) and ^18^F-Flutemetamol (Vizamyl™) for acquiring amyloid brain PET images. A total of 24 imaging centers, including 24 PET/CT and 4 PET/MR systems, were involved in acquiring brain PET images. Harmonization of PET images in this study was not performed prospectively due to the necessity of including previously existing amyloid PET images from parent cohorts acquired with historical image reconstruction protocols. Different reconstruction methods such as ordered subset expectation maximization (OSEM), line of response row action maximum likelihood algorithm (LOR-RAMLA), basis function ordered subset algorithm (BLOB-OS) with and without utilization of time of flight (TOF) and/or point spread function modeling (PSF) were used for reconstructing clinical amyloid PET images. The system model and reconstruction parameters for each site are shown in Table 1.

**Table 1:** Summary of scanner model and image reconstruction settings for each scanner

Manufacturer	S-ID	Model		R-ID	Reconstruction setting					Post-smoothing filter
					Algorithm	TOF	PSF	I × S	Voxel size (mm^3^)	FWHM (mm)
GE	1	Discovery MI	PET/CT	1	OSEM	No	No	102	2.00 × 2.00 × 2.79	All-pass
	2	Discovery MI	PET/CT	2	OSEM	No	No	102	2.00 × 2.00 × 2.79	All-pass
	3	Discovery MI	PET/CT	3	OSEM	No	No	96	2.00 × 2.00 × 2.79	All-pass
	4	Discovery MI	PET/CT	4	OSEM	No	No	102	2.00 × 2.00 × 2.79	All-pass
	5	Discovery 690	PET/CT	5	OSEM	No	No	72	1.00 × 1.00 × 3.27	All-pass
	6	Discovery 690	PET/CT	6	OSEM	No	No	180	1.17 × 1.17 × 3.27	All-pass
	6	Discovery 690	PET/CT	7	OSEM	No	No	180	1.17 × 1.17 × 3.27	4.50
Philips	7	Vereos	PET/CT	8	LOR-RAMLA	Yes	No	63	2.00 × 2.00 × 2.00	All-pass
	8	Ingenuity TF	PET/CT	9	BLOB-OS-TF	Yes	No	99	2.00 × 2.00 × 2.00	–
	9	Ingenuity TF	PET/MR	10	LOR-RAMLA	No	No	8	2.00 × 2.00 × 2.00	–
	10	Gemini TF 16	PET/CT	11	LOR-RAMLA	Yes	No	99	2.00 × 2.00 × 2.00	All-pass
Siemens	11	Biograph mCT	PET/CT	12	OSEM	No	No	96	1.02 × 1.02 × 2.03	All-pass
	12	Biograph 6 True Point	PET/CT	13	OSEM	No	No	84	1.02 × 1.02 × 2.03	All-pass
	13	Biograph mCT Flow	PET/CT	14	OSEM	No	No	96	1.02 × 1.02 × 2.03	All-pass
	14	Biograph 6 True Point	PET/CT	15	OSEM	No	No	64	1.01 × 1.01 × 2.00	All-pass
	15	Biograph 128 mCT	PET/CT	16	OSEM	No	No	96	1.02 × 1.02 × 2.03	All-pass
	16	Biograph 6 True Point	PET/CT	17	OSEM	No	No	64	1.01 × 1.01 × 2.00	All-pass
	17	Biograph128 mCT	PET/CT	18	OSEM	No	No	96	1.02 × 1.02 × 2.03	All-pass
	18	Biograph mCT Flow	PET/CT	19	OSEM	No	No	96	1.02 × 1.02 × 2.03	All-pass
	19	Biograph mCT	PET/CT	20	OSEM	No	No	96	1.02 × 1.02 × 2.03	All-pass
	20	Biograph mCT	PET/CT	21	OSEM	No	No	96	1.02 × 1.02 × 2.03	All-pass
	21	Biograph mCT	PET/CT	22	OSEM	No	No	96	1.02 × 1.02 × 2.03	All-pass
	22	Biograph mCT	PET/CT	23	OSEM	No	No	96	1.02 × 1.02 × 2.03	All-pass
	22	Biograph mCT	PET/CT	24	OSEM	Yes	Yes	168	1.02 × 1.02 × 2.03	3.00
	23	Biograph 40 TruePoint	PET/CT	25	OSEM	No	No	84	1.02 × 1.02 × 2.03	All-pass
	23	Biograph 40 TruePoint	PET/CT	26	OSEM	No	No	84	1.02 × 1.02 × 2.03	4.50
	24	Biograph	PET/MR	27	OSEM	No	No	96	1.04 × 1.04 × 2.03	All-pass
	25	Biograph 2	PET/CT	28	OSEM	No	No	64	1.33 × 1.33 × 3.37	All-pass
	26	Biograph mCT Flow 128 Edge	PET/CT	29	OSEM	Yes	No	96	1.02 × 1.02 × 2.03	All-pass
	27	Biograph mCT Flow 128 Edge	PET/CT	30	OSEM	Yes	No	48	1.59 × 1.59 × 2.00	All-pass
	28	Biograph mMR	PET/MR	31	OSEM	No	No	84	1.40 × 1.40 × 2.03	All-pass

It should be noted that for some scanners, two reconstruction settings were used corresponding to the AMYPAD and historical reconstruction protocols

*S-ID* system ID, *R-ID* reconstruction ID, *I × S* iterations × subsets, *OSEM* ordered subset expectation maximization, *TOF* time of flight, *PSF* point spread function modeling, *BLOB-OS* spherically symmetric basis function ordered subset algorithm, *RAMLA* row action maximum likelihood algorithm. System ID nos. 1, 2, and 4 consist of 4 rings with an axial field of view of 198 mm. System no.3 is a GE Discovery MI with 5 rings with an axial field of view of 249 mm

### AMYPAD image harmonization protocol

The harmonization protocol is based on the acquisition of a Hoffman 3D brain phantom and the calculation of the optimal Gaussian smoothing kernel to achieve a target image resolution. To this end, the protocol consists of two different sections including (1) a standardized operational procedure (SOP) for phantom image acquisition and (2) image preprocessing steps and calculation of image quality indicators. The SOP was developed to minimize data acquisition variability across different sites and was distributed among the imaging sites. It includes stepwise guidelines for phantom selections, phantom preparations, data acquisition details, reconstructions, and image restoration (Additional file 1). The second section of the harmonization protocol covers the calculations of the quality indicators by comparing the acquired phantom scan to the corresponding mathematical digital reference object to obtain the FWHM of the harmonization kernels for each site.

### Phantom image acquisition

A Hoffman 3D brain phantom was used in conjunction with a cylindrical pool phantom to simulate out-of-FOV (field of view) radioactive scatter from the body. The Hoffman phantom is constructed to provide an anatomically accurate simulation of brain regions and designed to simulate a 4:1 Gy matter to white matter activity concentration ratio observed in ^18^F-FDG brain PET scans of healthy subjects [26]. The Hoffman phantom was filled from a 1.5 L solution of ~ 18.5 MBq ^18^F-FDG, with a concentration of ~ 12.3 kBq/ml at the start of the PET scan. The cylindrical pool phantom had an internal diameter of ~ 16 cm and a length of 30 cm and was filled with ~ 80 MBq ^18^F-FDG solution. The Hoffman phantom was positioned at the center of the field of view, and the pool phantom was positioned 30–40 cm from the end of the Hoffman phantom. All emission data were acquired and reconstructed based on the clinical protocol used for AMYPAD amyloid brain PET scans across all involved imaging sites. It should be noted that Hoffman phantom data acquisition was different for 4 PET/MR systems included in this study as PET/MR systems have vendor-specific methods to calculate attenuation maps. For creating attenuation-corrected phantom PET images in this work, µ-maps were generated either using a CT image of the Hoffman phantom acquired on the same day on a CT scanner or using proper MR sequence on a cylindrical phantom filled with the saline solution. Detailed information is provided in the SOP (Additional file 1).

### Calculation of quality indicators

#### Image processing

Processing of the acquired phantom PET images was done within three different stages using SPM12. Data processing included steps for defining a site-specific digital reference object (DRO) using the scan information, estimating EIR of each system, defining the coarsest resolution, and estimating the FWHM of corresponding post-smoothing Gaussian kernel to achieve similar resolution across different sites.

#### Site-specific digital reference object (DRO)

Site-specific DRO was defined using the Hoffman phantom template VOI developed by Verwer et al. [18], representing gray matter, white matter, and ventricle compartments of the Hoffman phantom. Due to differences in matrix size and phantom positioning for each site, the phantom template was co-registered to the Hoffman PET image using rigid registration. Next, it was resliced to the PET dimension. Finally, site-specific DRO was created by assigning actual activity concentration at the beginning of the PET scan to gray matter and white matter VOIs (Additional file 2: Figure_S1). The activity concentration of ^18^F-FDG solution at starting PET scan was calculated based on two different methods:

1. *Dose-calibrator measurement* Measured syringe activity (kBq) after decay correction at scan time was divided by the volume of the water used for filling the phantom.
2. *Image-derived activity* First, GM and WM voxels of site-specific DRO were set to 1 and 0.25, respectively. In the next step, DRO was smoothed with an isotropic Gaussian filter with FWHM of 8 mm (s_8_DRO). Then, voxels with values above 0.98 were considered representative of the actual activity concentration of GM VOI (Pure-GM), as they were minimally affected by the smoothing and signal degradation due to the partial volume effect. Then, image-derived activity concentration was calculated by averaging activity over Pure-GM VOI in the PET image (Additional file 2: Figure_S2).

These two measurements were used to assess the calibration between the dose calibrator and the PET system, by computing their ratio. Based on EARL criteria [20], values of this ratio between 0.9 and 1.1 (± 10%) fall within the acceptable range. Finally, to avoid introducing instrument-related errors in the analysis, site-specific DRO was created by assigning image-derived activity concentration to GM and WM VOIs.

#### Estimating effective image resolution (EIR)

To estimate EIR, site-specific DRO was smoothed using an isotropic Gaussian filter (S_k_) with FWHM ranging from 0.1 to 10 mm, with an intercept of 0.1, resulting in 100 smoothed DRO (S_k_DRO).

1 $$\begin{gathered} \,S_{1} = 0.1\;{\text{mm}}; \hfill \\ \,S_{k} = S_{k} + 0.1; \, k = 1:100 \hfill \\ \,{\text{DRO}}_{k} = {\text{ DRO}} \otimes S_{k} \hfill \\ \end{gathered}$$

where DRO is the site-specific digital reference object, *S*_*k*_ is the *k*th Gaussian smoothing kernel, and DRO_*k*_ is the *k*th smoothed DRO and ⊗ is the convolution operator. The difference image was calculated by subtracting the phantom PET image from each smoothed DRO (Eq. 2). *S*_*k*_ was defined as EIR where the mean absolute global difference (GD) in DiffImage_**k**_ was minimum (Additional file 2: Figure_S3).

2 $${\text{DiffImage}}_{k} = {\text{ PET }} - {\text{ DRO}}_{k}$$

#### Estimating harmonization kernel

**Fig. 1:**
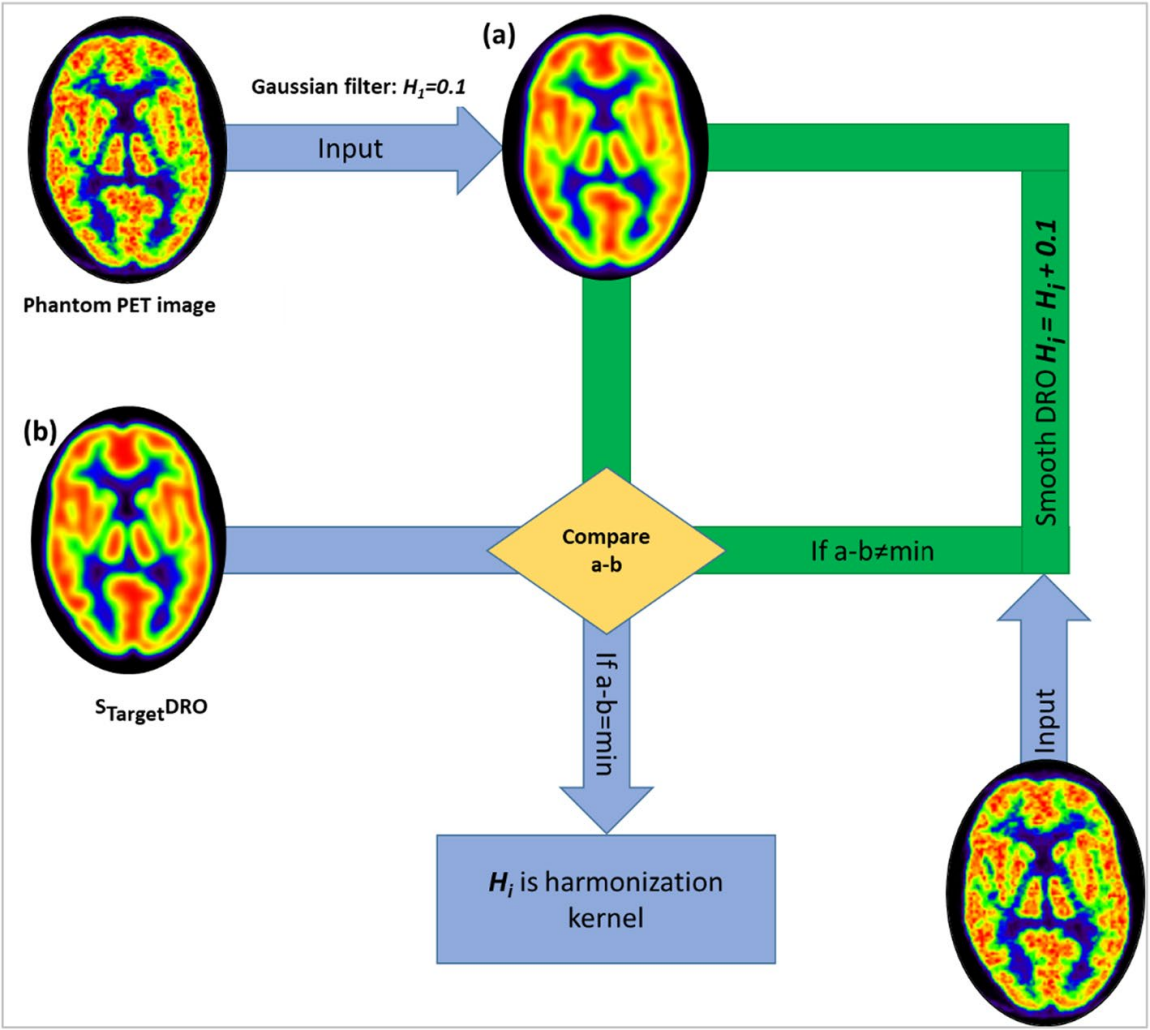
Schematic of iterative steps for estimating harmonization kernel to achieve 8-mm effective image resolution per site

The coarsest EIR was selected as target EIR for harmonizing the PET images. First, to determine the harmonization kernel for each site, site-specific DRO was smoothed with an isotropic Gaussian filter with FWHM equal to target image resolution (*S*_Target_DRO). In the next step, the phantom PET image was smoothed with 3D isotropic Gaussian filters (FWHM: *H*_*1*_ = 0.1 mm, *H*_*i*_ = *H*_*i*_ + 0.1, *i* = 1:100). Finally, the harmonization kernel (H_i_) was determined where the mean absolute global difference between smoothed PET image and *S*_Target_DRO was minimal (Fig. 1).

To illustrate that the method can also be used prospectively to achieve sharper target resolution, the procedure was repeated to achieve 6-mm target EIR in a subset of scans with 4–6-mm EIR.

#### Quantification of image quality indicators

To evaluate the performance of the harmonization protocol, different image quality indicators were extracted from the Hoffman phantom PET images before and after harmonization using the “Hoffman 3D Brain Phantom Analysis Tool” [18]. The quantitative image quality indicators were described and calculated as follows:

- Global and regional gray matter recovery coefficient (GMRC) as below:
  3 $${\text{GMRC}} = \frac{{{\text{Mean}}\;{\text{Activity}}\;{\text{ concentration}}\;{\text{at}}\;{\text{ GM }}\;{\text{ROI}}}}{{{\text{Actual}}\;{\text{ activity}}\;{\text{ concentration}} }}\quad {\text{GMRC}} \le 1$$

Additionally, GMRC was calculated for eroded GM VOI (GMRC_erod_), using the mask defined in the toolbox. This mask was defined with the purpose to minimize the partial volume effect on RC values [18].

- Contrast
  4 $${\text{Contrast}} = \frac{{{\text{Global}}\;{\text{ mean}}\;{\text{ activity}}\;{\text{ concentration}}\;{\text{ in}}\; {\text{GM VOI}}}}{{{\text{Global }}\;{\text{mean}}\; {\text{activity}}\;{\text{ concentration}}\;{\text{ in}}\; {\text{WM VOI}}}}\quad {\text{Contrast}} \le 4$$

Contrast was calculated using the GM and WM VOIs as well as eroded GM and WM (Contrast_erod_).

- Coefficient of variance (COV%) as an indicator of image homogeneity in a uniform background was calculated for five circular VOIs drawn on the WM (or semi-oval VOI)
  5 $${\text{COV}}\% = \frac{{{\text{Standard}}\;{\text{ deviation}}\;{\text{of}}\;{\text{activity}}\;{\text{concentration}}\;{\text{in}}\;{\text{ROI}}}}{{{\text{Mean}}\;{\text{ of}}\;{\text{activity }}\;{\text{concentration}}\;{\text{ in}}\;{\text{ ROI}}}} \times 100$$
- Left to right hemisphere RC ratio was calculated by dividing GMRC of the right hemisphere GM VOI to the left side. Ideally, this value should be 1, confirming the symmetrical performance of software and hardware of PET systems.
- Cold-spot recovery coefficient (cold-spot RC) was calculated for a predefined VOI in the midphantom simulating brain region with zero uptake.

### Sensitivity analysis

The performance of the harmonization protocol was assessed under different conditions. A sensitivity analysis was conducted to evaluate the impact of under/overestimation of the activity concentration of site-specific DRO on estimating EIR and harmonization kernel. To this end, first, a systematic ± 10% error was added to the calculated image-derived activity of DRO. Then, the EIR and harmonization kernel were estimated for error-induced DROs following the steps mentioned earlier and compared to the values obtained without introducing this error.

### Statistical analysis

The performance of the proposed method on harmonizing PET images was evaluated using different statistical criteria. Achieving a COV ≤ 15% and contrast ≥ 2.2 for eroded GM and WM VOIs was considered as acceptable image quality criteria, as recommended previously [19]. The percentage of systems with acceptable image quality was assessed before and after the harmonization procedure. In addition, the mean, standard deviation, and interquartile range (IQR) of quantification metrics (GMRC, contrast, and COV%) were calculated across imaging sites and compared before and after harmonization.

The stability and robustness of the harmonization output were tested by introducing dose-calibrator errors into the analysis. Comparison between EIR and harmonization kernel after introducing ± 10% error was made using Bland–Altman plot. Intraclass correlation coefficient (ICC) was used to estimate the consistency between EIR and harmonization kernels before and after inducing a ± 10% error.

## Results

### Acquisition of phantom scans

**Fig. 2:**
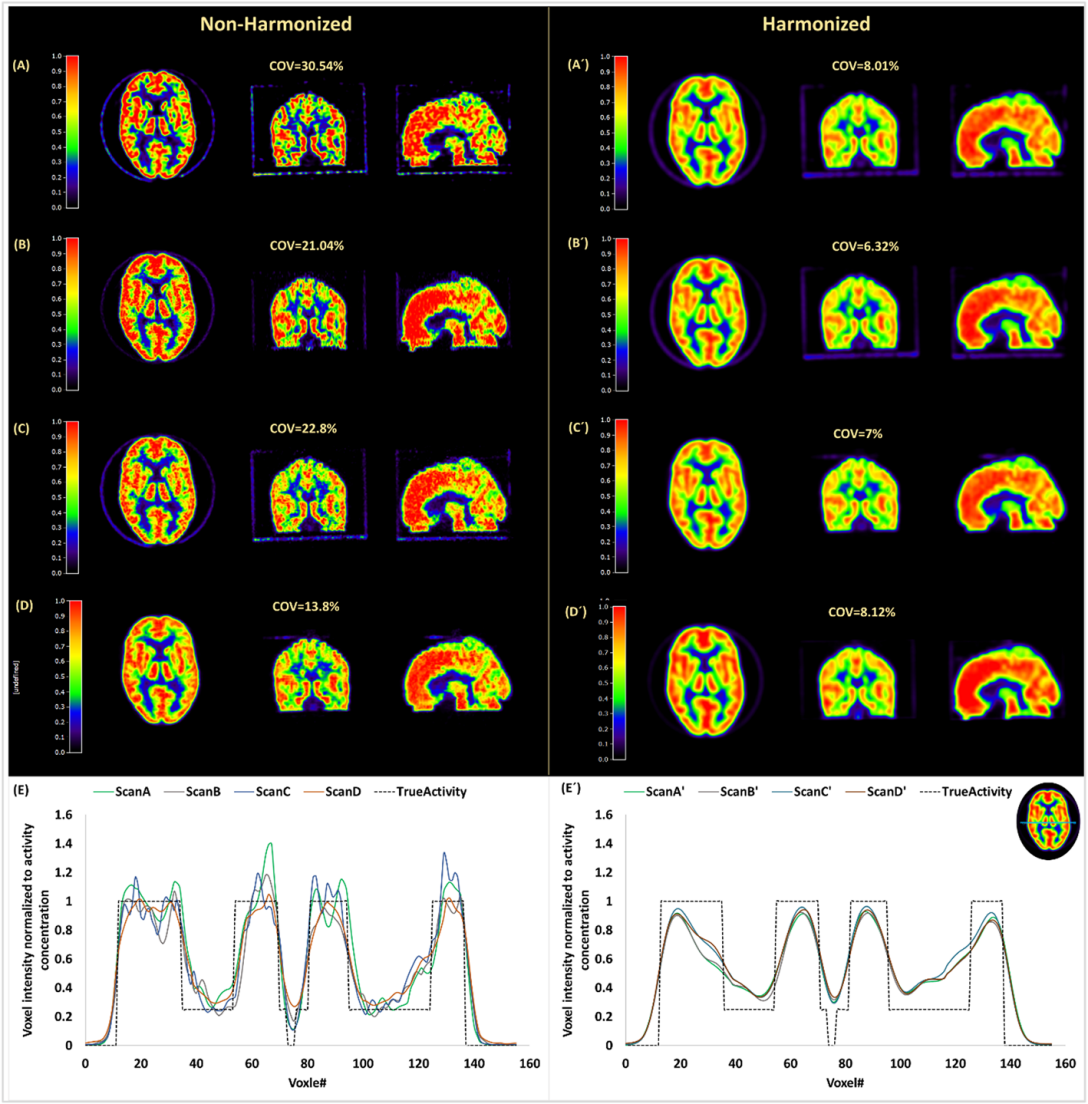
Visual comparison of phantom PET images acquired across 4 different sites before (**A**–**D**) and after (**A**′–**D**′) harmonization. Panels **E** and **E**′ represent corresponding line profiles before and after harmonization, respectively. All images are normalized to the image-derived activity

A total of 31 PET images, reconstructed using either AMYPAD or historical reconstructions, were included and analyzed. The left panels of Fig. 2 show examples of original (un-harmonized) phantom PET images acquired on four different PET systems. Hoffman phantom emission data were acquired following AMYPAD image harmonization SOP in 22 systems (67% of the total). The rest (*n* = 11; 33% of the total) had available Hoffman phantom acquisitions which were used for harmonization. These acquisitions only used the Hoffman phantom which was filled with ~ 40 MBq of ^18^F-FDG, resulting in a concentration of ~ 35 kBq/mL at the beginning of the PET scan.

**Fig. 3:**
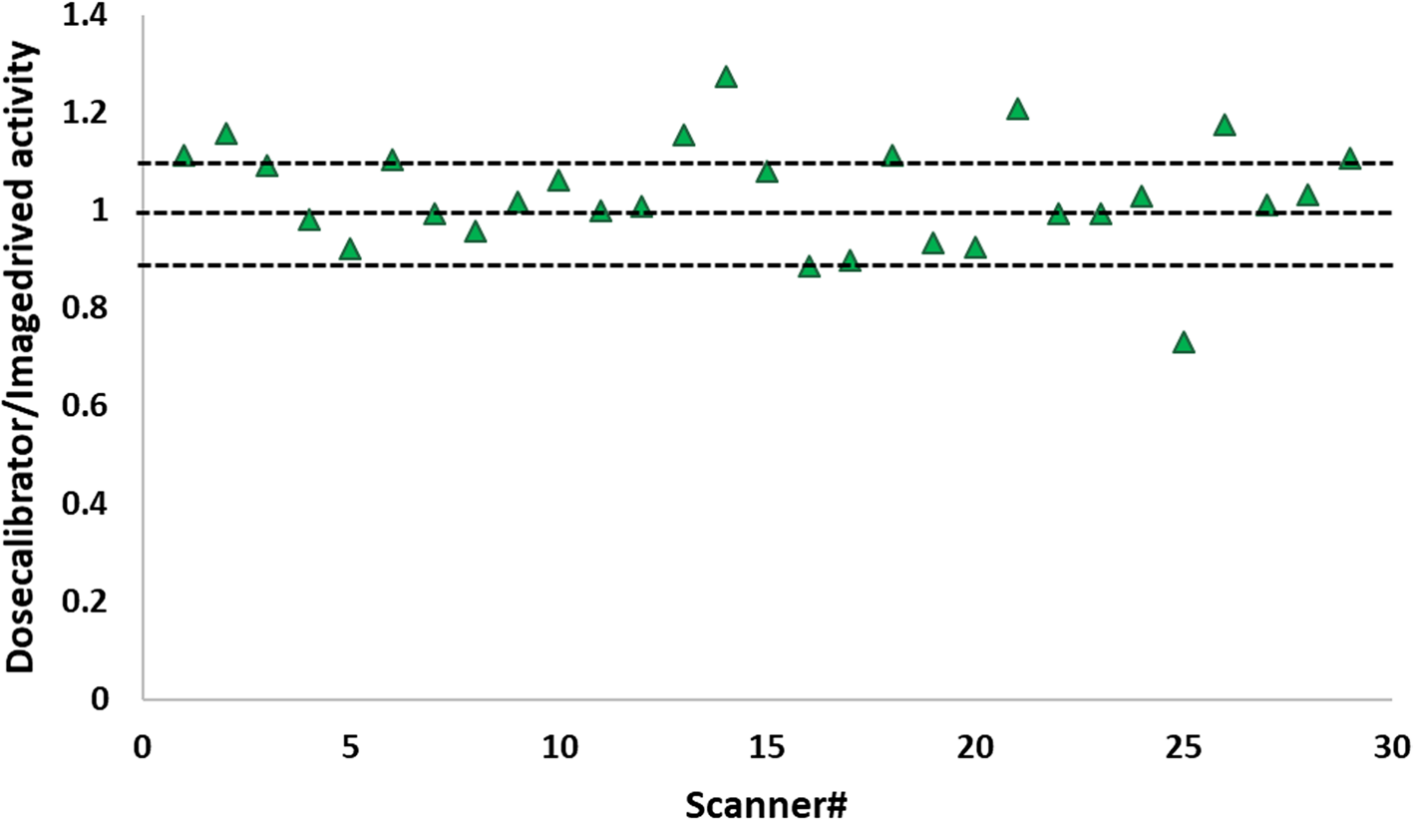
The ratio of dose-calibrator-based activity concentration to image-derived activity concentration across different PET systems

Discrepancies between dose-calibrator-based activity and image-derived activity concentration are shown in Fig. 3. A mean of 1.05 ± 0.12 observed for the dose calibrator to image-derived activity ratio. The ratio fell between 0.9 and 1.1 for 58.62% of the systems, which meets the acceptable level of error (± 10%) based on EARL criteria [20]. However, the highest observed ratio was 1.27 and the lowest one was 0.73 (error level ~  ± 30%) for two different systems. Therefore, to avoid incorporating dose-calibrator errors in the analysis, site-specific DRO was defined using image-derived activity for all the analyses included in the results. The image-derived activity was 11.93 ± 3.66 MBq/mL for the phantom scans acquired using pre-defined AMYPAD protocol and 26.31 ± 6.34 MBq/mL for the phantom scans acquired using local protocols.

### Effective image resolution and harmonization kernel

Table 2 shows the estimated effective resolution (EIR) and corresponding harmonization kernel for each reconstructed scan acquired within the AMYPAD imaging network. The coarsest EIR was 8.1 mm, whereas the lowest observed EIR was 3.2 mm (Mean ± SD: 5.38 ± 1.15). As a result, 8 mm was selected as the target EIR for harmonizing the PET images across different systems. The lowest FWHM of the harmonization kernel was 3 mm, assigned to a PET image with an EIR of 7.1 mm, whereas the highest FWHM was 8 mm applied to a PET image with an EIR of 3.2 mm.

**Table 2:** Estimated effective image resolution and post-smoothing harmonization kernel for each reconstruction setting implemented across AMYPAD sites

R-ID	Reconstruction setting				Post-smoothing filter	Effective image resolution	Harmonization kernel
	Algorithm	TOF	PSF	I × S	FWHM (mm)	FWHM (mm)	FWHM (mm)
1	OSEM	No	No	102	All-pass	5.40	6.30
2	OSEM	No	No	102	All-pass	5.00	6.10
3	OSEM	No	No	96	All-pass	5.50	6.80
4	OSEM	No	No	102	All-pass	4.60	6.00
5	OSEM	No	No	72	All-pass	5.50	5.20
6	OSEM	No	No	180	All-pass	4.00	6.30
7	OSEM	No	No	180	4.50	6.50	4.50
8	LOR-RAMLA	Yes	No	63	All-pass	4.40	6.60
9	BLOB-OS-TF	Yes	No	99	–	5.00	6.30
10	LOR-RAMLA	No	No	8	–	6.10	5.00
11	LOR-RAMLA	Yes	No	99	All-pass	7.30	2.00
12	OSEM	No	No	96	All-pass	3.70	7.10
13	OSEM	No	No	84	All-pass	4.80	6.40
14	OSEM	No	No	96	All-pass	4.30	6.50
15	OSEM	No	No	64	All-pass	4.70	6.20
16	OSEM	No	No	96	All-pass	5.10	5.80
17	OSEM	No	No	64	All-pass	6.60	4.60
18	OSEM	No	No	96	All-pass	5.90	6.10
19	OSEM	No	No	96	All-pass	4.80	6.60
20	OSEM	No	No	96	All-pass	4.70	5.40
21	OSEM	No	No	96	All-pass	4.30	6.80
22	OSEM	No	No	96	All-pass	4.30	6.40
23	OSEM	No	No	96	All-pass	5.20	6.20
24	OSEM	Yes	Yes	168	3.00	3.20	9.20
25	OSEM	No	No	84	All-pass	5.90	6.00
26	OSEM	No	No	84	4.50	8.10	–
27	OSEM	No	No	96	All-pass	3.80	7.10
28	OSEM	No	No	64	All-pass	7.30	3.50
29	OSEM	Yes	No	96	All-pass	5.80	6.00
30	OSEM	Yes	No	48	All-pass	6.40	5.10
31	OSEM	No	No	84	All-pass	4.40	6.70

*R-ID* reconstruction ID, *I × S* iterations × subsets, *OSEM* ordered subset expectation maximization, *TOF* time of flight, *PSF* point spread function modeling, *BLOB-OS* spherically symmetric basis function ordered subset algorithm, *RAMLA* row action maximum likelihood algorithm

The EIR of 21 PET scans was between 4 and 6 mm, and these scans were harmonized to achieve 6-mm target EIR.

### Image quality indicators

Before harmonization, contrast_erod_ was between 2.2 and 4 for 31 scans (93%), where only thirteen PET scans (39.4%) had COV% below 15% and complying with acceptable image quality criteria. After harmonization, COV% and contrast_erod_ met acceptable quantitative criteria. Harmonizing PET images produced comparable visual image quality irrespective of the PET system model and technical differences (Fig. 2, right panels and Additional file 2: Figure_S5). Corresponding line profiles across scans are shown in Fig. 2E before harmonization. All scans were normalized to the activity concentration at the scan time. It should be noted that scan A was reconstructed using PSF modeling, resulting in voxel intensities above 1 due to Gibbs artifact, and high noise level, however after applying harmonization kernel, normalized voxel intensities across different scans became comparable within hot and cold regions (Fig. 2E′).

**Fig. 4:**
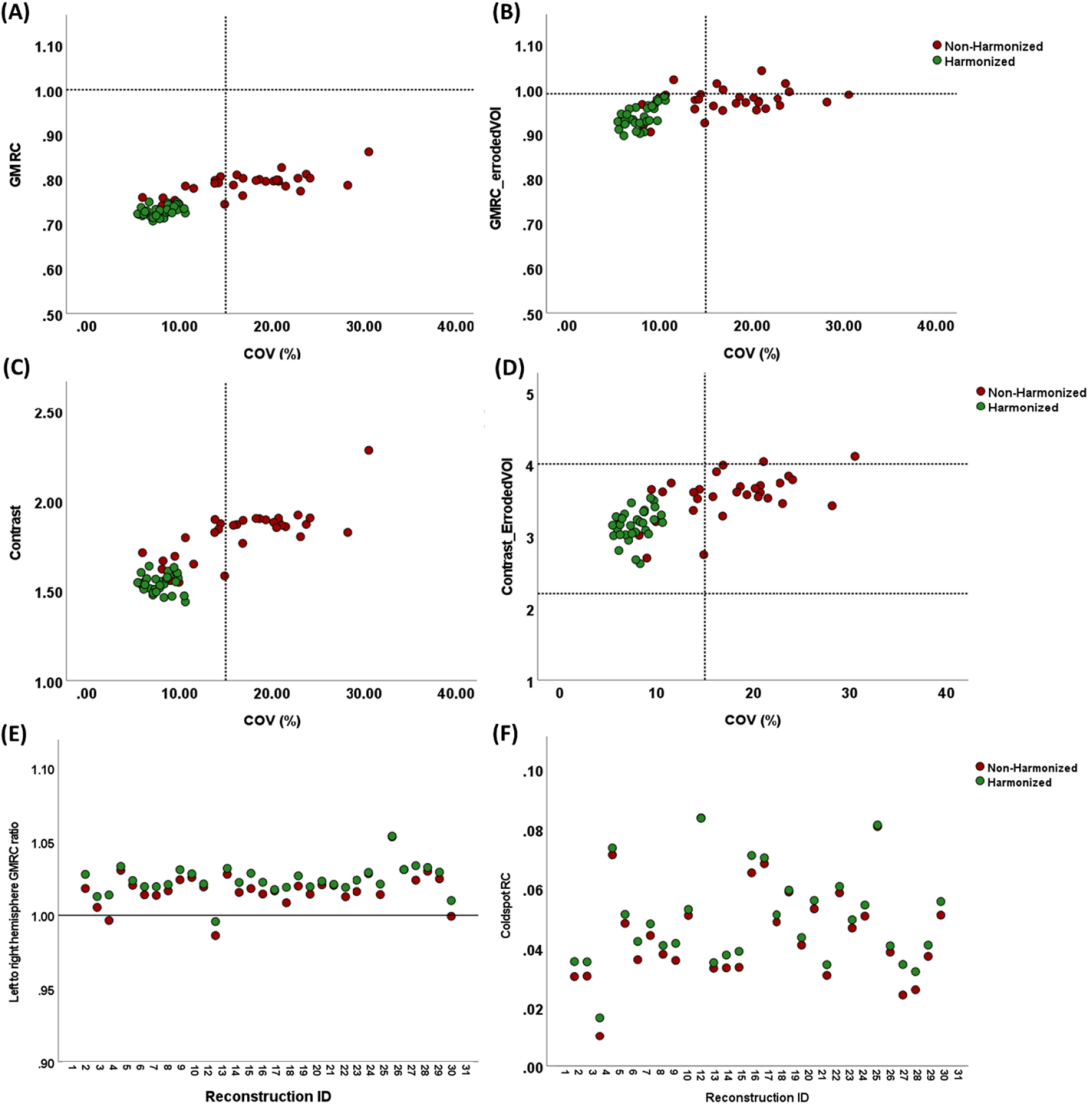
Comparing GMRC and contrast for **A**, **C** non-eroded and **B**, **D** eroded VOIs as a function of COV% for harmonized versus non-harmonized PET images. Panels **E**, **F** display left-to-right hemisphere RC ratios and cold-spot RC of different reconstructions for harmonized vs. non-harmonized images

Table 3 compares the different image quality indicators before and after harmonization. The inter-system variability of all quantification metrics (COV%, GMRC, and contrast) decreased significantly after matching EIR across different PET scans. The harmonization of PET images resulted in reducing the mean and spread of the COV% from 16.97 ± 6.03 to 7.86 ± 1.47%. A similar pattern was observed while harmonizing scans to 6-mm EIR (Additional file 2:Table_S1). The interquartile range of COV% decreased from 9.5% (median: 16.83%) to 2.65% (median: 7.88%). A mean contrast of 1.81 ± 0.14 was observed across different scans before harmonization, while harmonizing PET images reduced contrast variability (Mean ± SD: 1.54 ± 0.05). The same pattern was observed for GMRC, and the interquartile ranges were 0.040 (median: 0.790) and 0.012 (median: 0.725) before and after harmonization, respectively. The impact of the harmonization on the GMRC and contrast of non-eroded and eroded VOIs as a function of COV% was evaluated (Fig. 4 and Additional file 2: Figure_S6). Four PET scans (12%) showed a GMRC_erod_ above 1 before harmonization where GMRC_erod_ ≤ 1 was observed for all PET harmonized scans. GMRC_erod_ changed slightly from 0.97 ± 0.03 (IQR: 0.03) to 0.94 ± 0.02 (IQR: 0.03) after harmonization. However, contrast_erod_ was more affected by harmonization, and a mean contrast_erod_ of 3.53 ± 0.32 (IQR: 0.36) was observed for PET images before harmonization, and the mean value decreased to 3.06 ± 0.04 (IQR: 0.27) after harmonizing dataset. Left-to-right GMRC ratio was not affected by harmonization; however, variability across different systems was reduced as a result of decreasing standard deviation from 0.01 to 0.009 (Mean ± SD: 1.02 ± 0.01 and 1.02 ± 0.009 before and after harmonization, respectively). An absolute difference of 0.003 ± 0.002 was observed for cold-spot RC pre- and post-harmonization (Fig. 6E).

**Table 3:** Image quality metrics measured by the toolbox before and after harmonization

Quantification metrics	Non-harmonized Mean ± SD (range)	Harmonized Mean ± SD (range)
COV%	16.96 ± 6.03 (6–30.55)	7.88 ± 1.49 (5.49–10.64)
GMRC	0.78 ± 0.03 (0.74–0.86)	0.72 ± 0.01 (0.71–0.75)
Contrast	1.82 ± 0.14 (1.55–2.28)	1.54 ± 0.05 (1.44–1.64)
GMRC eroded VOI	0.97 ± 0.03 (0.90–1.04)	0.94 ± 0.02 (0.89–0.98)
Contrast eroded VOI	3.54 ± 0.32 (2.69–4.11)	3.16 ± 0.04 (2.62–3.53)
Left–right hemisphere GM RC ratio	1.02 ± 0.01 (0.99–1.05)	1.02 ± 0.009 (1.00–1.05)
Cold-spot RC	0.04 ± 0.01 (0.01–0.08)	0.05 ± 0.01 (0.02–0.08)

### Sensitivity analysis

**Fig. 5:**
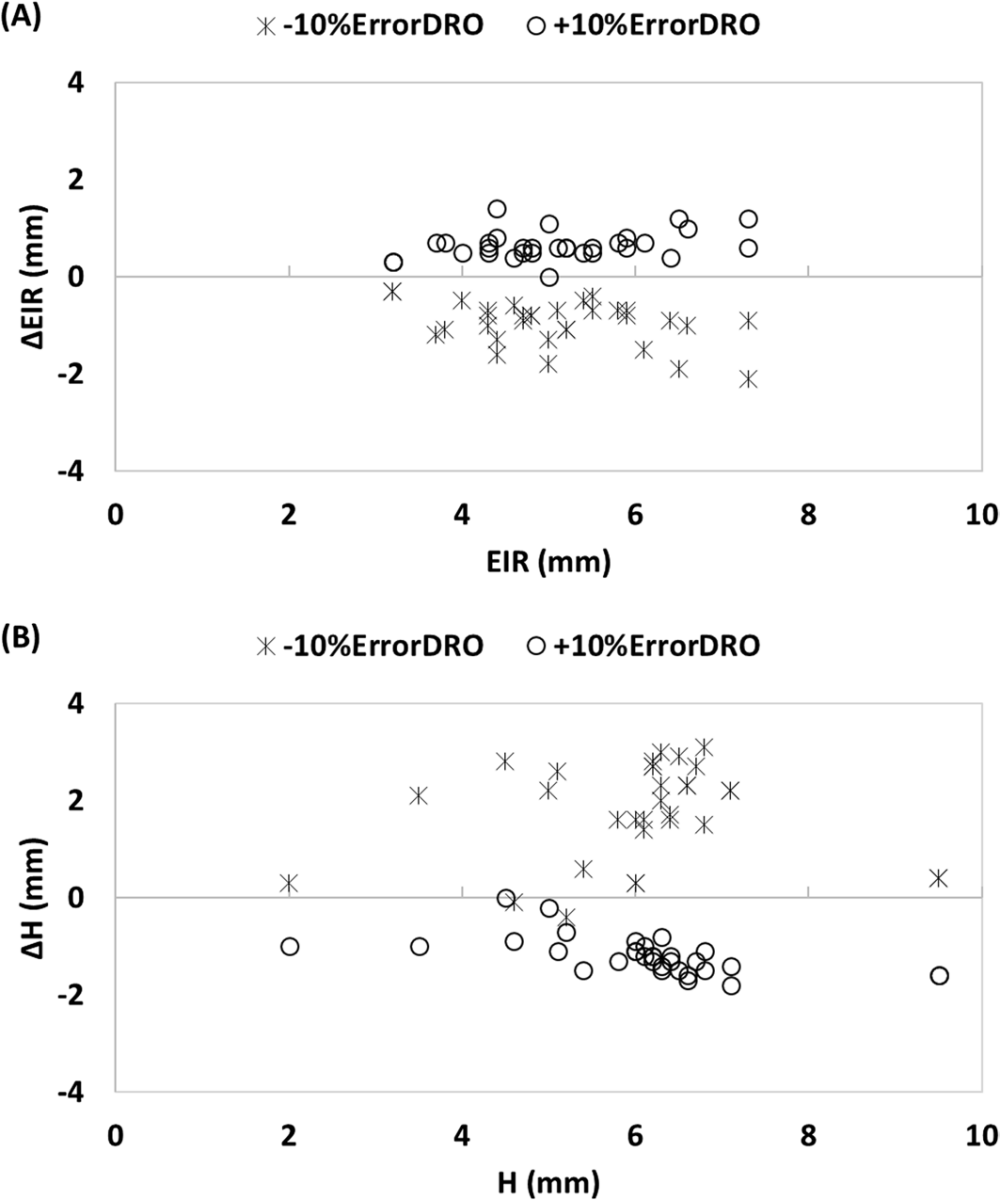
Bland–Altman plots comparing the difference between effective image resolution (**A**) and harmonization kernel (**B**) after introducing ± 10% error to DRO activity

Figure 5A shows the difference between estimated EIR after applying ± 10% error in site-specific DRO activity concentration. A 10% underestimation of the DRO activity resulted in an average underestimation of EIR below 1 mm (FWHM Mean ± SD: 0.96 ± 0.49 mm; 95% CI 0.80–1.12 mm). This error subsequently led to an increase below 2 mm in the harmonization kernel (FWHM Mean ± SD: 1.77 ± 0.95 mm 95% CI 1.43–2.11) to achieve the target EIR. On the other hand, an overestimation of the + 10% of the site-specific DRO activity led to an increase of 0.65 ± 0.28 mm in EIR (95% CI 0.55–0.75) and a decrease of 1.19 ± 0.39 mm (95% CI 1.05–1.32) in the harmonization kernel. The ICC was 0.96 (95% CI 0.93–0.98, *p* < 0.0001) for EIR and 0.93 (95% CI 0.88–0.96, *p* < 0.0001) for harmonization kernel before and after applying ± 10% error.

## Discussion

In this work, we assessed a framework for the harmonization of brain PET images using Hoffman phantom scans and the EIR as harmonization criterion. Using this method, we showed the feasibility of attaining PET scans of comparable quality, as assessed quantitatively and qualitatively, in multi-site imaging networks. Our complementary results showed that this method can be used prospectively for harmonizing scans acquired on state-of-the-art PET systems to target sharper EIR. The results confirmed that our proposed harmonization protocol is robust against dose-calibrator errors. Indeed, we were able to validate the proposed framework across different systems including PET/MR systems which pose challenges for harmonization due to their specific attenuation correction methods. Despite this, our method produced comparable quantitative metrics between PET/MR and PET/CT systems.

We showed that EIR is a robust criterion for harmonizing brain PET images acquired in different centers. EIR is a global metric comparing the simulated theoretical activity concentration per voxel in DRO using different Gaussian filters with the experimental activity concentration in the phantom PET image. In other words, EIR is the FWHM of a three-dimensional Gaussian filter that provides the best fit between the theoretical and experimental activity concentration values. One of the advantages of using EIR is including all voxels in the calculation of EIR, therefore taking into account simultaneously the signal degradation, image uniformity, and spill-in and spill-out between different compartments of the Hoffman phantom (representing GM, WM, and ventricles). This characteristic makes EIR a robust parameter for image harmonization as it is not sensitive to the presence of small bubbles, slight shape differences of the Hoffman phantoms, or dose-calibrator errors. We additionally considered EIR as a symmetrical Gaussian filter with the same FWHM in *x*, *y* (trans-axial), and *z* (axial) directions, meaning that the resolution of a PET system in the axial direction is higher than radial and tangential resolutions. However, it is reasonable to assume uniform smoothing in brain imaging as the patient is located in the center of the system where the field of view is fairly uniform in all directions. As the coarsest estimated EIR was around 8 mm across different PET scans, the target EIR was selected to be 8 mm. Harmonizing all PET images to the target EIR was performed by smoothing PET images with better spatial resolution. This allowed for reducing the standard deviation of COV% across different PET images as well as the COV% to the acceptable level (COV ≤ 15%) (Table 3). It should be noted that for the data with sharper EIR, COV% has higher values and higher levels of variabilities are observed across different scans with similar EIRs. As a consequence, most of the images do not meet the acceptance COV% for achieving optimal image quality as COV% falls above 15% in most of them (Additional file 2: Figure_S5).

In this study, contrast, GMRC, COV%, cold-spot RC, and left-to-right GMRC ratio were used as complementary indicators for evaluating the performance of the harmonization method. Our results indicated that mapping EIR across different systems produced comparable quantitative image quality metrics irrespective of the system model and reconstruction setting (Table 3 and Fig. 4). It should be noted that in the current work, 4 PET/MR systems were included in the harmonization procedure, and we were able to validate the feasibility of harmonization of PET/MR systems in multi-center studies. Contrast and GMRC have been previously recommended for harmonizing brain PET images in multi-center studies [18, 19]. While keeping contrast and GMRC between the lower and upper acceptance range helps to achieve optimal image quality and reduce variabilities, it does not necessarily result in harmonized images. The main reason for the insufficient performance of contrast and GMRC as harmonization criteria comes from the way these metrics are defined. Contrast is calculated as the ratio of mean activity in gray matter VOI to the mean activity concentration in white matter VOI, meaning that the noise property will be canceled out by this division. GMRC is the mean activity concentration in the GM mask divided by true activity at the starting PET scan, where the GM mask is big enough to minimize the noise effect. In other words, it is possible to achieve PET images with similar contrast and GMRC, but with different noise levels (Fig. 4). Another limitation of using contrast and GMRC as harmonization metrics is the way GM and WM VOIs are defined. For example, some studies used eroded WM and GM VOIs for extracting contrast and GMRC. The main disadvantage of that approach is that quantitative metrics extracted from eroded VOIs are based on the limited number of voxels. That is, using small VOIs for quantification can be sensitive to noise and it normally represents the part of the image that is minimally affected by signal degradation and partial volume effect. However, the main aim of harmonizing PET images is to acquire the same level of signal degradation across different systems. According to our results on quantification of un-harmonized PET images, fifteen PET scans (44.11%) were complying with contrast and GMRC limits for both eroded and non-eroded VOIs recommended by Verwer et al. However, COVs% ranging from 10.64 to 28.19% (18.09 ± 4.50%) were observed for these PET scans, showing a high level of heterogeneity, confirming that contrast and GMRC are not efficient enough for reducing between scanner variabilities. After harmonizing PET images, although quantitative metrics variabilities were reduced significantly, none of these metrics were complying with the recommended limits [18]. In a previous study, the acceptance limits for quantitative criteria were defined by using optimized reconstruction protocols, meaning that these limits cannot be applicable to the scans reconstructed with protocols deviating from optimal reconstruction and those historical scans that have been acquired previously, using older systems.

**Fig. 6:**
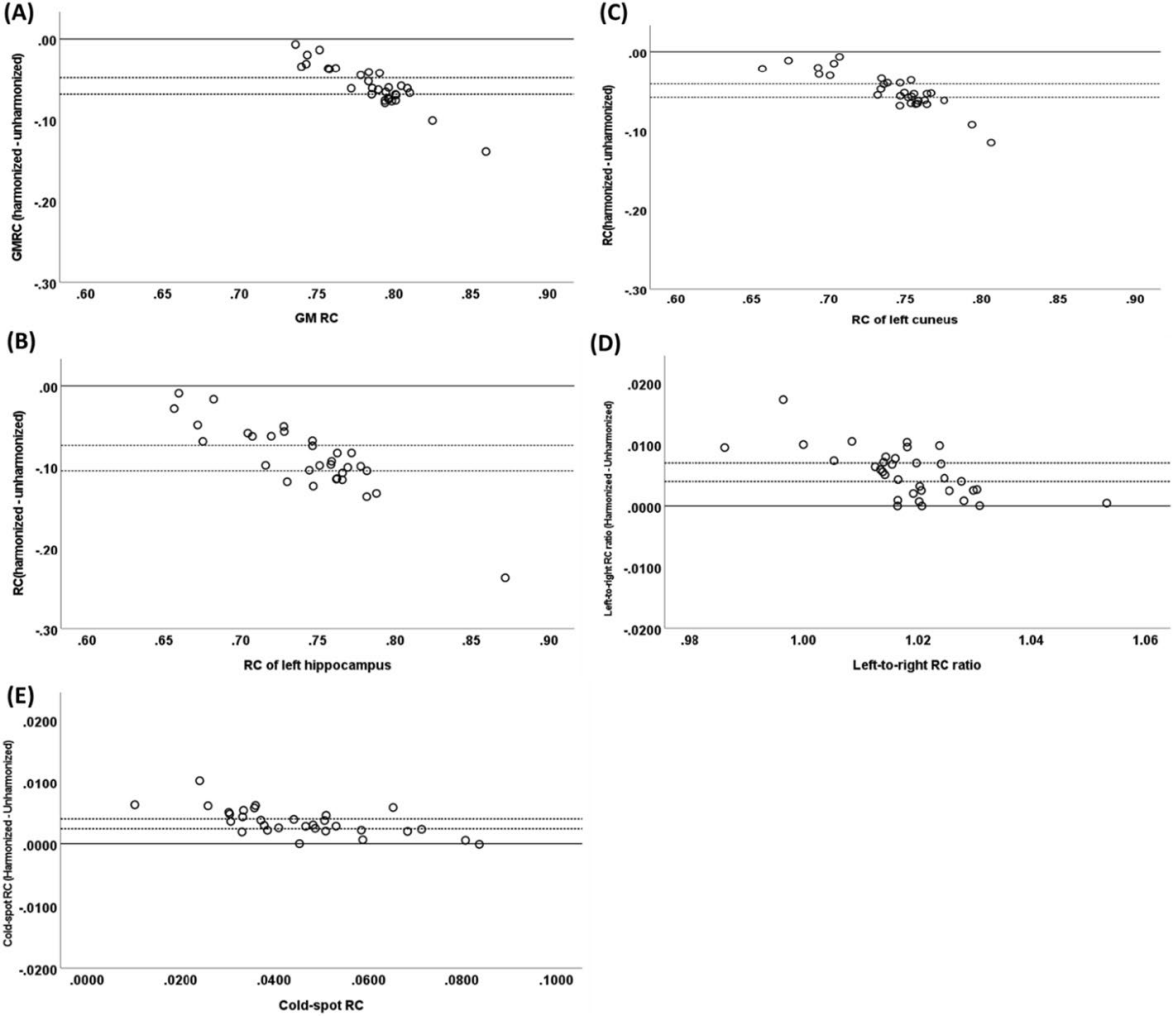
Bland–Altman plots comparing differences between RCs for **A** global GM, **B** left hippocampus, **C** left cuneus, **D** left-to-right RC ratio, and **E** cold-spot RC before and after harmonization. Dashed lines represent 95% confidence intervals, and solid lines represent the line of equality

RC of cold VOIs (regions with low uptake) is an important quantitative metric, and its accuracy depends on the spatial resolution as well as scatter correction algorithms [27]. Since scatter correction algorithms are different across different vendors, they could produce different values in low uptake VOIs. In this study, we evaluated the accuracy of cold-spot RC before and after harmonization. As cold-spot was defined in a VOI without uptake, a recovery coefficient of zero was expected. According to our results, cold-spot RCs were very close to zero for the majority of the centers (Mean: 0.04 95% CI 0.04–0.05), confirming similar performance of scatter corrections across different vendors and PET system models. However, a cold-spot RC of 0.08 was observed for one PET/MR system which could be due to the errors of generated attenuation correction map in PET/MR phantom scan. Uniformity of the PET images across the field of view was measured using left-to-right GMRCs. Given that the system should provide uniform performance across the FOV, a left-to-right GMRC ratio of 1 is assumed for all systems. In this study, the left-to-right GMRC ratio was in the expected range and no significant difference was observed among different systems (1.02 ± 0.01, 95% CI 1.01–1.02). Additionally, as expected, harmonizing PET images had minimal effect on the mean cold-spot RC (Mean ± SD difference: 0.003 ± 0.002) as well as right-to-left hemisphere GMRC ratios (Mean ± SD difference: 0.005 ± 0.004) (Fig. 6). Although the highest acceptable value for GMRC is one, we observed GMRC_erod_ > 1 for five of the un-harmonized PET data. Similar behavior was observed for contrast_erod_, where contrast_erod_ > 4 was observed for two PET scans. This level of overestimation can be explained by either a high level of noise propagation or the presence of Gibbs artifact due to reconstruction settings. However, after harmonizing PET images, GMRC_erod_ (0.89–0.98) and contrast_erod_ (2.62–3.53) fell below the acceptable level, which is due to minimizing the noise effect or correcting Gibbs artifact after applying post-smoothing filter (Table 3).

Our results on the performance of the harmonization method on quantitative metrics confirmed that the proposed methodology is capable of harmonizing PET data and producing comparable quantitative metrics across different PET images (Fig. 4). Despite the capacity of this methodology in minimizing differences in PET data, one potential caveat could be that smoothing the PET images to the poorest EIR decreases its sensitivity in detecting small changes. However, present results indicated that by harmonizing the images, global GMRC was minimally reduced 0.06 ± 0.03 after harmonization and maintained good recovery coefficients for both large VOIs such as cuneus as well as small VOIs such as the hippocampus (Fig. 6) while drastically reducing the COV% spread across sites. The highest decrease between the quantitative metrics before and after harmonization was observed for a PET image reconstructed with PSF modeling, and global RC and left cuneus RC decreased by − 0.12, and left hippocampus RC reduced by − 0.24 after harmonization of the PET images. The reason behind this significant decrease is that due to the reconstruction settings significant Gibbs artifact was observed, resulting in overshooting the signal, especially in the small VOIs like the hippocampus, and harmonization of these data improved image quality by removing the Gibbs artifact as well as producing comparable images across different centers.

Different levels of error between dose-calibrator estimated activity and image-derived activity concentration were observed. Our results showed that dose-calibrator and image-derived activity concentration ratio is between 0.73 and 1.27 across the AMYPAD imaging network, representing the overestimation/underestimation of activity concentrations across different centers (Fig. 3). These discrepancies can be explained by different sources of error such as errors in phantom preparation and estimating stock volume solution, calibration errors, or quality control errors of the dose calibrator. Introducing a ± 10% error in the image-derived activity to the DRO for estimating EIR and harmonization kernel had a negligible impact on both EIR (~ 1 mm) and estimated harmonization kernel (~ 1.5 mm), confirming that our methodology in estimating EIR and harmonization kernel is stable and robust enough to the expected level of errors (Fig. 5). Image-derived activity is a suitable substitute for dose-calibrator activity to avoid including dose-calibrator-based errors in the harmonization procedure. However, to calculate image-derived activity, it is important to include only voxels in the gray matter that are far from the edge of the GM mask, to avoid any possible contribution of the Gibbs artifact in the case of using point spread function modeling in the reconstruction. Additionally, voxels that are minimally or not affected by partial volume effect and signal degradation should be used for estimating image-derived activity. In this framework, DRO was smoothed using a Gaussian filter with FWHM of 8 mm and only voxels with intensity above 0.98 were considered as pure gray matter for calculating image-derived activity. The reason behind selecting this filter is that the coarsest EIR of a commercial PET system is about 8 mm, and to avoid underestimation of image-derived activity, it is necessary to include voxels that are minimally affected by PVE across all available PET systems. Also, voxels with intensity above 0.98 are far from the edge of GM VOI and eliminated the possibility of over-estimating activity due to Gibbs artifact.

In this study, the brain PET harmonization framework was implemented in two clinical trials of AMYPAD, and our results confirmed the feasibility of using this method for both PET/CT and PET/MR systems. Most of the imaging centers in AMYPAD network had EARL accreditation, and a NEMA phantom scan has been conducted across most imaging sites for evaluating the quantitative performance of the system before starting clinical PET scans. As a result, the level of variabilities in the AMYPAD imaging network is probably lower compared to general clinical settings. Based on our framework, true activity concentration was calculated using a data-driven metric, without any need for cumbersome preprocessing steps. Estimating the EIR of each PET system was done using the DRO provided by a software tool developed for the automated analysis of Hoffman PET images. Using this toolbox, only phantom PET image and scan info were introduced as the inputs, and many image quality and quantitative metrics were extracted as outputs. This toolbox is developed for automated analysis of Hoffman phantom PET images and enabled us to calculate several quantitative metrics of phantom PET using a consistent framework for evaluating the PET quantitative criteria across different centers before and after harmonization.

## Strengths and limitations

This framework can be implemented in multi-center studies for the harmonization of brain PET images. Even though the Hoffman phantom is designed in a way to reproduce the ^18^F-FDG uptake in the brain, the harmonization methodology can be generalizable to other tracers used for brain PET imaging. EIR is a product of the reconstruction constant (*β*_reconstruction_) and FWHM of the system [28], defined as below:

6 $$\begin{aligned} & {\text{EIR}} = \, \beta_{{{\text{reconstruction}}}} \times {\text{FWHM}}_{{{\text{system}}}} \\ & {\text{FWHM}}_{{{\text{system}}}} = \sqrt {\left( \frac{d}{2} \right)^{2} + \left( {0.0022D} \right)^{2} + r^{2} } \\ \end{aligned}$$

where *d* and *D* correspond to detector size and ring diameter, and *r* is the positron range. According to Eq. 6, the EIR of a specific reconstruction acquired on a PET system is the same for other tracers with similar positron ranges. In the case of using tracers like ^11^C-PiB, the Hoffman phantom should be filled with the specific tracer and the entire harmonization framework is applicable for calculating EIR and harmonization kernel. It should be noted that EIR could be slightly different for tracers with different positron ranges.

One limitation of this framework is that this methodology is not capable of reducing variability in very low uptake regions and further research is needed. Another limitation of this study is that due to the presence of historical reconstructions, harmonization of the data was done retrospectively, and PET images were smoothed in a way to match the coarsest EIR. Although the main argument against smoothing data to the coarsest resolution is losing sensitivity, our results showed that harmonization had a minimal impact on the GMRC and contrast. The proposed framework can harmonize the PET components of the hybrid PET systems. Therefore, concerning the PET/MR systems, it is important to note that due to differences between CT-based and MR-based attenuation correction of clinical PET images, their quantification will be different from the phantom setting during the harmonization or accreditation procedure. Our proposed framework can be implemented in a prospective study for the harmonization of PET images. To harmonize the data in a prospective study, it is suggested to choose reconstruction settings in a way to meet acceptance quantitative criteria as well achieving a predefined EIR. Applying this framework in a prospective study can be useful to achieve optimal reconstruction settings and harmonization simultaneously. The feasibility and performance of this proposed method in prospective data harmonization should be tested in future studies.

## Conclusions

In this study, we introduced and validated a harmonization framework for producing brain PET scans of comparable quality across a wide variety of PET systems in the imaging network of real-world clinical trials. Also, this method can be used prospectively to harmonize the scans acquired on new systems with sharper image resolution. Various quantitative and qualitative metrics were extracted from PET images before and after harmonization by a software toolbox for automated analysis of the Hoffman 3D Phantom. EIR is a valid metric for harmonizing PET images as it resulted in comparable COV%, GMRC, and contrast across sites and was robust against dose-calibrator-based errors. Additionally, cold-spot RC and left-to-right GMRC ratio were stable before and after harmonization. Using the proposed framework, it is feasible to harmonize brain PET data prospectively as well as retrospectively for commercially available PET/MR and PET/CT systems. Prospective harmonization of the data could be combined in a way to achieve acceptable limits for GMRC, and contrast, as well as achieving similar EIR across different systems. This procedure needs to be tested on the prospective data.

### Supplementary Information

**Additional file 1**. Standard Operational Procedure (SOP) for AMYPAD harmonization project, providing the description of the necessary steps for obtaining Hoffman phantom scan for PET/CT and PET/MRI systems.

**Additional file 2**. Figures and tables show the schematic steps for preprocessing the Hoffman phantom PET images, estimating effective image resolution, harmonization Kernel, and image quality metrics.

## Data Availability

The data that support the findings of this study are available from the corresponding author upon request.
