# Supplementary material for: Harmonization of brain PET images in multi-center PET studies using Hoffman phantom scan
Source: EJNMMI Phys. 2023 Oct 31;10:68. doi: 10.1186/s40658-023-00588-x (PMC10618151; doi:10.1186/s40658-023-00588-x)
Supplement: Supplementary file 1 — Additional file 1. Standard Operational Procedure (SOP) for AMYPAD harmonization project, providing the description of the necessary steps for obtaining Hoffman phantom scan for PET/CT and PET/MRI systems. [file 40658_2023_588_MOESM1_ESM.docx]

***Standard Operational Procedure for AMYPAD harmonization project***

Contents

[Standard Operational Procedure 2](#_Toc144202371)

[*SOP for PET/CT systems* 3](file:///D:\Harmonizationdaraft\SOP_Brain_PET_Harmonization_Amypad_Reviewer.docx#_Toc144202372)

[Hoffman Phantom Protocols for Harmonization Project for PET/CT systems 4](#_Toc144202373)

[Materials: 4](#_Toc144202374)

[Preparing the Phantoms 4](#_Toc144202375)

[Preparing the pool Phantom: 4](#_Toc144202376)

[Preparing the Hoffman phantom 5](#_Toc144202377)

[Data Acquisition 6](#_Toc144202378)

[Filling the scan report form 8](#_Toc144202379)

[Emptying phantom 8](#_Toc144202380)

[*SOP for PET/MR systems* 9](file:///D:\Harmonizationdaraft\SOP_Brain_PET_Harmonization_Amypad_Reviewer.docx#_Toc144202381)

[Hoffman Phantom Protocols for Harmonization Project for PET/MR systems 10](#_Toc144202382)

[Materials: 10](#_Toc144202383)

[Preparing the Phantoms 11](#_Toc144202384)

[Preparing the pool Phantom: 11](#_Toc144202385)

[Preparing the Hoffman phantom 11](#_Toc144202386)

[Preparing water phantom: 12](#_Toc144202387)

[Data Acquisition 12](#_Toc144202388)

[A. Using MR-based attenuation correction: 12](#_Toc144202389)

[B. Using CT-based template attenuation map: 14](#_Toc144202390)

[Filling the scan report form 16](#_Toc144202391)

[Emptying phantom 16](#_Toc144202392)

# Standard Operational Procedure

This document provides a comprehensive description of the necessary procedures for obtaining Hoffman phantom scans. As attenuation correction techniques are different between PET/MR and PET/CT systems and for the sake of clarity, distinct sections have been dedicated to outlining the steps specific to each system.

# *SOP for PET/CT systems*

# Hoffman Phantom Protocols for Harmonization Project for PET/CT systems

It should be noted that all the centers should have passed the quality control criteria for image quality and uniformity described in the quality assurance guidelines. Also, to avoid introducing biases, all the PET, CT, and dose calibrator clocks should be synchronized. In summary, the following points should be considered in each site:

- Dose calibrator should be accurate and has passed its QC in place
- Time on the imaging system should be synchronized with the time of the activity measurements (deviation less than 10 s)
- System should pass the daily QC on the day that phantom scan is going to be acquired, and there should not be any known issue with the system

# Materials:

- Personnel protective equipment including gloves, lab coat
- Hoffman brain phantom
- Pool phantom, a cylindrical phantom with 16 cm inner diameter (range 14-18 cm) and 30 cm length (range 25-30 cm)
- A container or bottle with a volume of 1500 mL that can be securely sealed
- A container with a volume of 50 mL
- 18.5 MBq (0.5 mCi), with a range of 17-20 MBq of ^18^F-FDG for the Hoffman phantom
- 80 MBq (2.16 mCi), with a range 75-85 MBq of ^18^F-FDG for the pool phantom
- 60 mL syringe and needles
- 10 mL syringes and needles
- 10 cm long catheter/line/tubing

# Preparing the Phantoms

# Preparing the pool Phantom:

- Fill the pool phantom with the taped or distilled water
- Fix the cover plate of the phantom using nylon screws
- Remove filler plugs and remove 10 to 15 mL of the water with a syringe
- Inject the prepared ^18^F-FDG syringe calibrated for having 80 MBq (2.16 mCi) at the time of acquisition into the phantom and flush the syringe several times to minimize the residual
- Close the phantom tightly to avoid any leakage and shake it to get a uniformly distributed ^18^F-FDG solution
- Reopen the filling port and add previously removed water to fill the phantom

# Preparing the Hoffman phantom

- Preparing the tracer in a syringe to get an activity of 18.5 MBq (0.5 mCi) of ^18^F-FDG at the intended beginning of the PET scan
- Mixing the tracer with taped or distilled water in another container with a volume of 1500 mL
- To minimize the residual inside the syringe, rinse the syringe a couple of times. It helps to wash out the radiotracer from the syringe and reduce the residual inside the syringe.
- Measuring the residual dose activity in the syringe by dose calibrator and time of measurement
- Homogenize the stock solution
- Removing cover plate from the Hoffman phantom and pour about 900 mL of the solution in the main compartment of the phantom
- Reposition and fix the cover plate of the phantom using the 6 nylon screws
- Invert the phantom a few times and shake it rotationally to remove the trapped bubbles between the plates and achieve uniform distribution of the ^18^F-FDG solution
- Remove one of the caps/filler plugs and try to fill the phantom with a 60 cc syringe with a catheter and needle.
- Try to fill the rest by sticking the needle in the first slide and leads

**Note: If there are bubbles that are not big and will not create a big photopenic area, it will not be problematic.

**Note: For the Hoffman phantom, it is recommended to use distilled water to prevent natural pollution such as algae, specially in the inner layers of the Hoffman phantom. This issue could happen when the phantom remains filled for extended periods, or if, after emptied, water becomes trapped in its inner layers.

# Data Acquisition

- Place an absorption pad on the couch covering both Hoffman and pool phantom to avoid any spillage
- Positioning the phantom in the center of the field of view, in a way that filling plugs are towards the feet of the bed. The anterior part of the phantom should be on the top, in the 12 o’clock position and the cerebellar (posterior) part should be positioned near the bed as shown in Figure 1(b). By positioning the phantom in this way, we can simulate a clinical PET scan in a supine position.

Figure1. Three-dimensional Hoffman Phantom (a), CT image of a Hoffman phantom showing the correct positioning in the field of view (b).


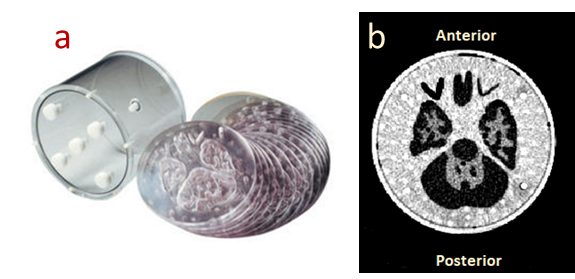


- Hoffman phantom should be placed in a holder on the bed or either be fixed using sponges in two sides of the phantom to avoid possible rolling during the scan (or use tape to fix it)
- Position the pool phantom next to the end of the Hoffman phantom on the bed out of the ﬁeld to simulate body activity
- Start the acquisition by entering the following data
- Weight of the Hoffman phantom: 1.2 kg
- Name acquisition: **HAMYPAD_SiteID**
- Syringe activity used for filling the Hoffman phantom
- Choose the acquisition protocol used for the clinical amyloid PET scan based on Site Operation Guide (SOG)
- Acquire the topogram CT and center the phantom in the field of view
- Acquire the CT attenuation scan for making the attenuation correction map. CT protocol including kv_p,_ mA, and tube rotation should be the same as the clinical amyloid PET scan in each center (Described on the SOG)
- Scan the Hoffman phantom for 30 minutes or more to acquire the PET data
- All PET data should be acquired in list mode
- PET images should be reconstructed based on the AMYPAD SOG which has been defined for each site based on the system model previously. In addition, for non-EPAD cohorts, PET data should be reconstructed with the historical clinical protocol that they already used for amyloid PET image reconstruction.
- If the time of flight or point spread function modeling is available on the system, PET data could be reconstructed with TOF, PSF, and TOF+PSF

** More detailed information for the reconstruction protocol will be provided for each site specifically by considering the system model and will be shared with the physicist before data acquisition.

- The voxel size of the images should be based on the sizes that have already been defined in AMYPAD SOG. For non-EPAD cohorts, the voxel size should be defined based on the historical protocol.
- For all above reconstruction, the following time frames should be reconstructed: 1x30 min, 6x5 min frames
- Put data on DVD /CD-ROM /etc. without removing important PET DICOM TAGS
- DICOM PET images should be saved in Bq/mL unit
- Saving the list mode data of the Hoffman acquisition, if possible, for possible reconstructions in future
- To avoid missing any data from the DICOM header, no anonymization should be done

# Filling the scan report form

All the required information in the scan report form could be provided by the physicist in charge of scanning the phantom. In this form the following information is needed:

- The value of the activity written on each syringe
- Measured activity in the dose calibrator and time of measuring the activity
- Time of the starting the scan
- Reconstruction protocol that has been used for the phantom including algorithm, number of iterations and subsets, type, and full width at half maximum of the post smoothing filter in all x y z directions
- Scan duration for each reconstruction
- Observations for reporting any possible issues that happened during the acquisition

# Emptying phantom

Emptying the phantom and transferring it to the radioactive waste storage room will be done following the local guidelines and regulations for radiation safety implemented in each imaging site.

# *SOP for PET/MR systems*

# Hoffman Phantom Protocols for Harmonization Project for PET/MR systems

It should be noted that all the centers should have passed the quality control criteria for image quality and uniformity described in the quality assurance guidelines. Also, to avoid introducing biases, all the system and dose calibrator clocks should be synchronized. In summary, the following points should be considered in each site:

- Dose calibrator should be accurate and has passed its QC in place
- Time on the system should be synchronized with the time of the activity measurements (deviation less than 10 s)
- System should pass the daily QC on the day that phantom scan is going to be acquired, and there should not be any known issue with the system

## Materials:

- Personnel protective equipment including gloves, lab coat
- Hoffman brain phantom
- Pool phantom, a cylindrical phantom with 16 cm inner diameter (range 14-18 cm) and 30 cm length (range 25-30 cm)
- A container or bottle with a volume of 1500 mL that can be securely sealed
- A container with a volume of 50 mL
- 18.5 MBq (0.5 mCi), with a range of 17-20 MBq of ^18^F-FDG for the Hoffman phantom
- 80 MBq (2.16 mCi), with a range 75-85 MBq of ^18^F-FDG for the pool phantom
- 60 mL syringe and needles
- 10 mL syringes and needles
- 10 cm long catheter/line/tubing
- 1 Liter of Saline solution (0.9%)
- Oil capsule
- Water phantom, a cylindrical phantom with the inner diameter equivalent to Hoffman phantom diameter (inner +plastic), preferably Jaszczak phantom (the best fitting)

# Preparing the Phantoms

## Preparing the pool Phantom:

- Fill the pool phantom with the taped or distilled water
- Fix the cover plate of the phantom using nylon screws
- Remove filler plugs and remove 10 to 15 mL of the water with a syringe
- Inject the prepared ^18^F-FDG syringe calibrated for having 80 MBq (2.16 mCi) at the time of acquisition into the phantom and flush the syringe several times to minimize the residual
- Close the phantom tightly to avoid any leakage and shake it to get a uniformly distributed ^18^F-FDG solution
- Reopen the filling port and add previously removed water to fill the phantom

## Preparing the Hoffman phantom

- Preparing the tracer in a syringe to get an activity of 18.5 MBq (0.5 mCi) of ^18^F-FDG at the intended beginning of the PET scan
- Mixing 750 mL of water with 750 mL of saline solution 0.9% in the container. Saline solution is very helpful for getting a uniform MR signal.
- Add 18.5 MBq (0.5 mCi) of ^18^F-FDG, close the container, and shake the container to get a uniform ^18^F-FDG solution.
- To minimize the residual inside the syringe, rinse the syringe a couple of times. It helps to wash out the radiotracer from the syringe and reduce the residual inside the syringe.
- Measuring the residual dose activity in the syringe by dose calibrator and time of measurement
- Homogenize the stock solution
- Removing cover plate from the Hoffman phantom and pour about 900 mL of the solution into the main compartment of the phantom
- Reposition and fix the cover plate of the phantom using the 6 nylon screws
- Invert the phantom few times and shake it rotationally to remove the trapped bubbles between the plates and achieve uniform distribution of the 18F-FDG solution
- Remove one of the caps/filler plugs and try to fill the phantom with a 60 cc syringe with a catheter and needle.
- Try to fill the rest by sticking the needle in the first slide and leads

**Note: If there are bubbles that are not big and will not create a big photopenic area, it will not be problematic.

**Note: For the Hoffman phantom, it is recommended to use distilled water to prevent natural pollution such as algae, specially in the inner layers of the Hoffman phantom. This issue could happen when the phantom remains filled for extended periods, or if, after emptied, water becomes trapped in its inner layers.

## Preparing water phantom:

Water phantom is used for the PET/MRI sites which it is feasible to get an attenuation map using the MRI sequence and introduce this MR-based estimated attenuation map for the attenuation correction of the PET data.

- Fill 50% of the water phantom body compartment with water and fill the rest with the saline solution 0.9%.
- Fix the cover plate of the phantom using nylon screws
- Close the phantom tightly to avoid any leakage

## Data Acquisition

## Using MR-based attenuation correction:

- Place an absorption pad on the couch covering both Hoffman and pool phantom to avoid any spillage
- Position the water phantom in the center of the field of view
- Start the acquisition by entering the following data
- Weight of the Hoffman phantom: 1.2 kg
- Name acquisition: **HAMYPAD_SiteID**
- Syringe activity used for filling the Hoffman phantom
- Choose the acquisition protocol used for the clinical amyloid PET scan based on SOG
- Setting proper MRI protocols for the phantom MR acquisition (i.e., setting homogeneity correction to none)
- Acquire the MRI scan to get the estimation of the attenuation map
- Swapping the phantoms, and positioning the Hoffman phantom in the same location as the cylindrical water phantom
- Positioning the Hoffman phantom in the center of the field of view, in a way that filling plugs are towards the feet of the bed. The anterior part of the phantom should be on the top, in the 12 o’clock position and the cerebellar (posterior) part should be positioned near the bed as shown in Figure 1(b). By positioning the phantom in this way, we can simulate a clinical PET scan in a supine position.
- Hoffman phantom should be placed in a holder on the bed or either be fixed using sponges on two sides of the phantom to avoid possible rolling during the scan (or use tape to fix it)
- Position the pool phantom next to the end of the Hoffman phantom on the bed out of the ﬁeld to simulate body activity
- Scan the Hoffman phantom for 30 minutes or more to acquire the PET data
- All PET data should be acquired in list mode
- PET images should be reconstructed based on the AMYPAD SOG which has been defined for each site based on the system model previously. In addition, for non-EPAD cohorts, PET data should be reconstructed with the historical clinical protocol that they already used for amyloid PET image reconstruction.
- If the time of flight or point spread function modeling is available on the system, PET data could be reconstructed with TOF, PSF, and TOF+PSF

** More details information for the reconstruction protocol will be provided for each site specifically by considering the system model and will be shared with the physicist before data acquisition

- Voxel size of the images should be based on the sizes that have already been defined in AMYPAD SOG. For non-EPAD cohorts the voxel size should be defined based on the historical protocol.
- Put data on DVD /CD-ROM /etc. without removing important PET DICOM TAGS
- Saving the list mode data of the Hoffman acquisition, if possible, for possible reconstruction in future
- To avoid missing any data from the DICOM header, no anonymization should be done

## Using CT-based template attenuation map:

This part is for the systems that allow us to use the attenuation map from a CT scan.

- Place an absorption pad on the CT couch to avoid any spillage
- Attach an oil capsule to the anterior part of the phantom
- Do not move the oil capsule till finishing the acquisition, because it is necessary for coregistering MRI and CT images in the reconstruction step
- Positioning the phantom in the center of the field of view, in a way that filling plugs are towards the feet of the bed. The anterior part of the phantom should be on the top, in the 12 o’clock position and the cerebellar (posterior) part should be positioned near the bed as shown in Figure 1(b). By positioning the phantom in this way, we can simulate a clinical PET scan in a supine position.
- Hoffman phantom should be placed in a holder on the bed or either be fixed using sponges in two sides of the phantom to avoid possible rolling during the scan (or use tape to fix it)
- Scan the phantom using the CT protocol described in the SOG
- Move the phantom to the PET/MR system couch
- Place an absorption pad on the PET/MR couch to avoid any spillage
- Position the Hoffman phantom in the center of the field of view, using the same instructions done for the CT
- Position the pool phantom next to the end of the Hoffman phantom on the bed out of the ﬁeld to simulate body activity
- Start the acquisition by entering the following data
- Weight of the Hoffman phantom: 1.2 kg
- Name acquisition: **HAMYPAD_SiteID**
- Syringe activity used for filling the Hoffman phantom
- Choose the acquisition protocol used for the clinical amyloid PET scan based on SOG
- Scan the Hoffman phantom for 30 min for acquiring the PET data
- All PET data should be acquired in list mode
- Coreregister CT image with the MR Dixon in phase image
- Converting the Hounsfield unit of the CT image to linear attenuation coefficient
- Resample the converted CT to the PET voxel size
- Reconstruct the PET images using this provided attenuation correction map
- PET images should be reconstructed based on AMYPAD SOG which has been defined for each site based on the system model previously. In addition, for non-EPAD cohorts, PET data should be reconstructed with the historical clinical protocol that they already used for amyloid PET image reconstruction.
- If the time of flight or point spread function modeling is available on the system, PET data could be reconstructed with TOF, PSF, and TOF+PSF

** More details information for the reconstruction protocol will be provided for each site specifically by considering the system model and will be shared with the physicist before data acquisition

- Voxel size of the images should be based on the sizes that have already been defined in AMYPAD SOG. For non-EPAD cohorts the voxel size should be defined based on the historical protocol.
- Put data on DVD /CD-ROM /etc. without removing important PET DICOM TAGS
- Saving the list mode data of the Hoffman acquisition, if possible, for possible reconstruction in future

## Filling the scan report form

All the required information in the scan report form could be provided by the physicist in charge of scanning the phantom. In this form following information is needed:

• The value of the activity written on each syringe

• Measured activity in the dose calibrator and time of measuring the activity

• Time of the starting the scan

• Reconstruction protocol that has been used for the phantom including algorithm, number of iterations and subsets, type and full width at half maximum of the post smoothing filter in all x y z directions

• Scan duration for each reconstruction

• Observations for reporting any possible issues that happened during the acquisition

# Emptying phantom

Emptying the phantom and transferring it to the radioactive waste storage room will be done following the local guidelines and regulations for radiation safety implemented in each imaging site.
