# Supplementary material for: Harmonization of brain PET images in multi-center PET studies using Hoffman phantom scan
Source: EJNMMI Phys. 2023 Oct 31;10:68. doi: 10.1186/s40658-023-00588-x (PMC10618151; doi:10.1186/s40658-023-00588-x)
Supplement: Supplementary file 2 — Additional file 2. Figures and tables show the schematic steps for preprocessing the Hoffman phantom PET images, estimating effective image resolution, harmonization Kernel, and image quality metrics. [file 40658_2023_588_MOESM2_ESM.docx]

**Harmonization of brain PET images in multi-center PET studies using Hoffman phantom scan**

Mahnaz Shekari^1-3^, Eline. E. Verwer^4^, Maqsood Yaqub^4^, Marcel Daamen^5^, Christopher Buckley^6^, Giovanni B. Frisoni^7,8^, Pieter Jelle Visser^9-12^, Gill Farrar^6^, Frederik Barkhof^4,13^, Juan Domingo Gispert^1-3,14*^, Ronald Boellaard^4*^, on behalf of the AMYPAD Consortium

^1^Barcelonaβeta Brain Research Center (BBRC), Pasqual Maragall Foundation. Barcelona, Spain

^2^IMIM (Hospital del Mar Medical Research Institute), Barcelona, Spain

^3^Universitat Pompeu Fabra, Barcelona, Spain

^4^Department of Radiology & Nuclear Medicine, Amsterdam, University Medical Centers, location VUmc, De Boelelaan 1117, 1081 HV Amsterdam, The Netherlands

^5^German Center for Neurodegenerative Diseases (DZNE), Bonn, Germany

^6^GE Healthcare, Amersham, United Kingdom

^7^Laboratory of Neuroimaging of Aging (LANVIE), University of Geneva, Geneva, Switzerland

^8^Memory Center, Department of Rehabilitation and Geriatrics, University Hospitals and University of Geneva Geneva, Switzerland

^9^Alzheimer Center Amsterdam, Neurology, Vrije Universiteit Amsterdam, Amsterdam UMC

location VUmc, Amsterdam, The Netherlands

^10^Amsterdam Neuroscience, Neurodegeneration, Amsterdam, The Netherlands

^11^Alzheimer Center Limburg, School for Mental Health and Neuroscience, Maastricht

University, Maastricht, the Netherlands

^12^Department of Neurobiology, Care Sciences and Society, Division of Neurogeriatrics, Karolinska Institutet, Stockholm Sweden

^13^Institute of Neurology and Centre for Medical Image Computing, University College London, UK

^14^Centro de Investigación Biomédica en Red Bioingeniería, Biomateriales y Nanomedicina, (CIBER-BBN), Barcelona, Spain

***Corresponding authors**


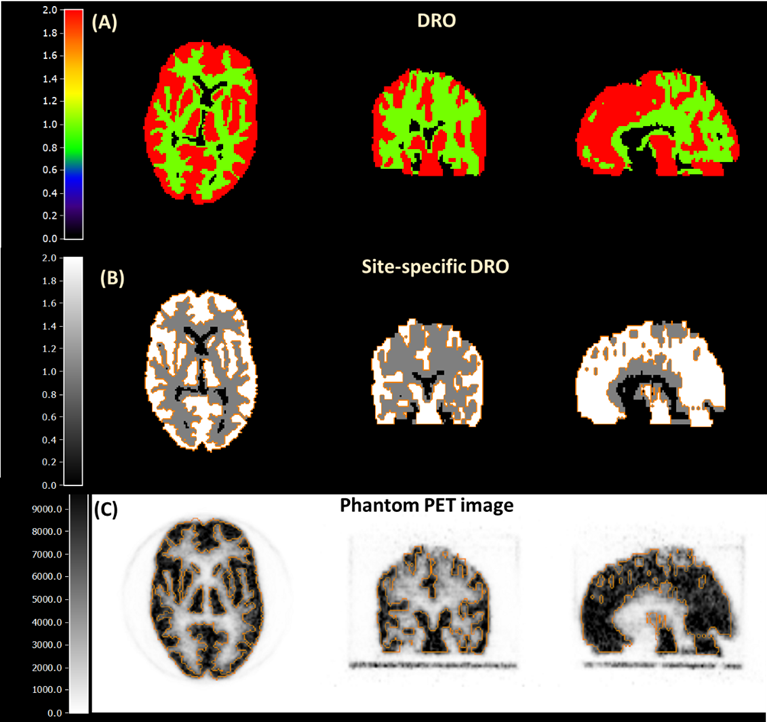


Figure_S1. Digital reference object (DRO) provided by toolbox (A) Site-specific DRO after realignment with PET scan (B) Example of PET phantom image with the overlaid contour of site-specific DRO.


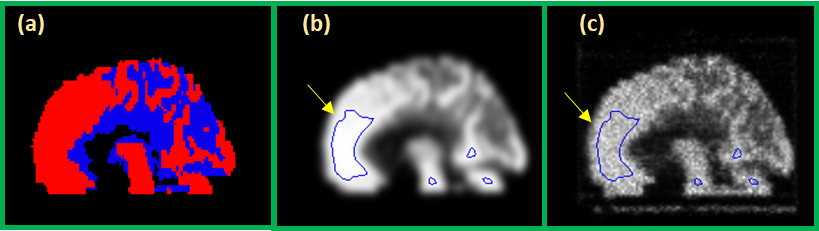


Figure_S2. Sagittal view of (A) digital reference object (B) smoothed DRO with Gaussian filter with an FWHM of 8 mm (S8-DRO), and (C) Hoffman phantom PET image. Blue counters in image (A) and (B) shows voxels with intensity above 0.98 and voxels included for calculating image-derived activity concentration, respectively.


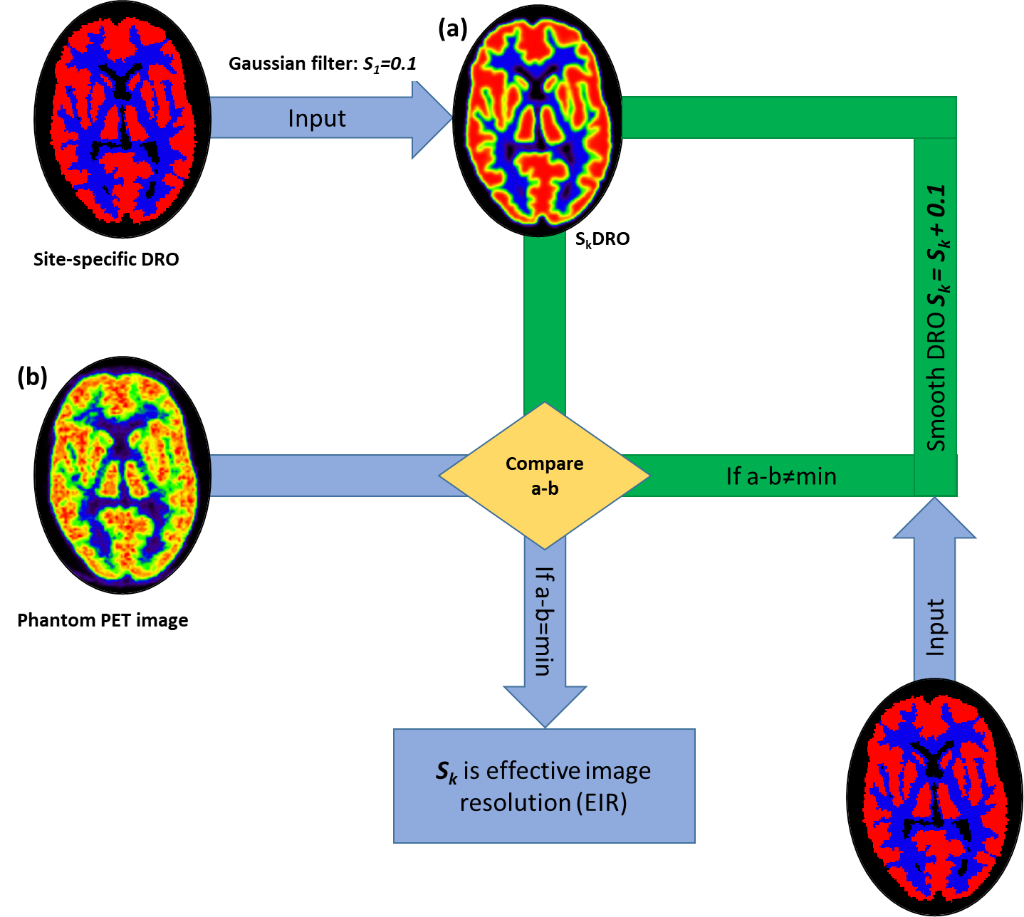


Figure_S3. Schematic of processing steps for estimating effective image resolution for each acquired phantom PET image.

Figure_S4 (A) shows the global mean absolute difference (across all voxels) between the acquired 3D Hoffman phantom and the DRO for smoothing kernels ranging from 0.1 to 10 mm FWHM as an example of the behavior of the method for estimating the EIR of a Siemens Biograph mCT 128 PET/CT scanner. The mean absolute global difference converged to a minimum for a Gaussian filter of 4 mm FWHM, which was determined as effective image resolution (EIR). The phantom PET image converged to the target effective image resolution while applying a post-smoothing Gaussian filter of 6.6 mm FWHM, where the mean global absolute difference reached the minimum value (Figure_S4 (B)).


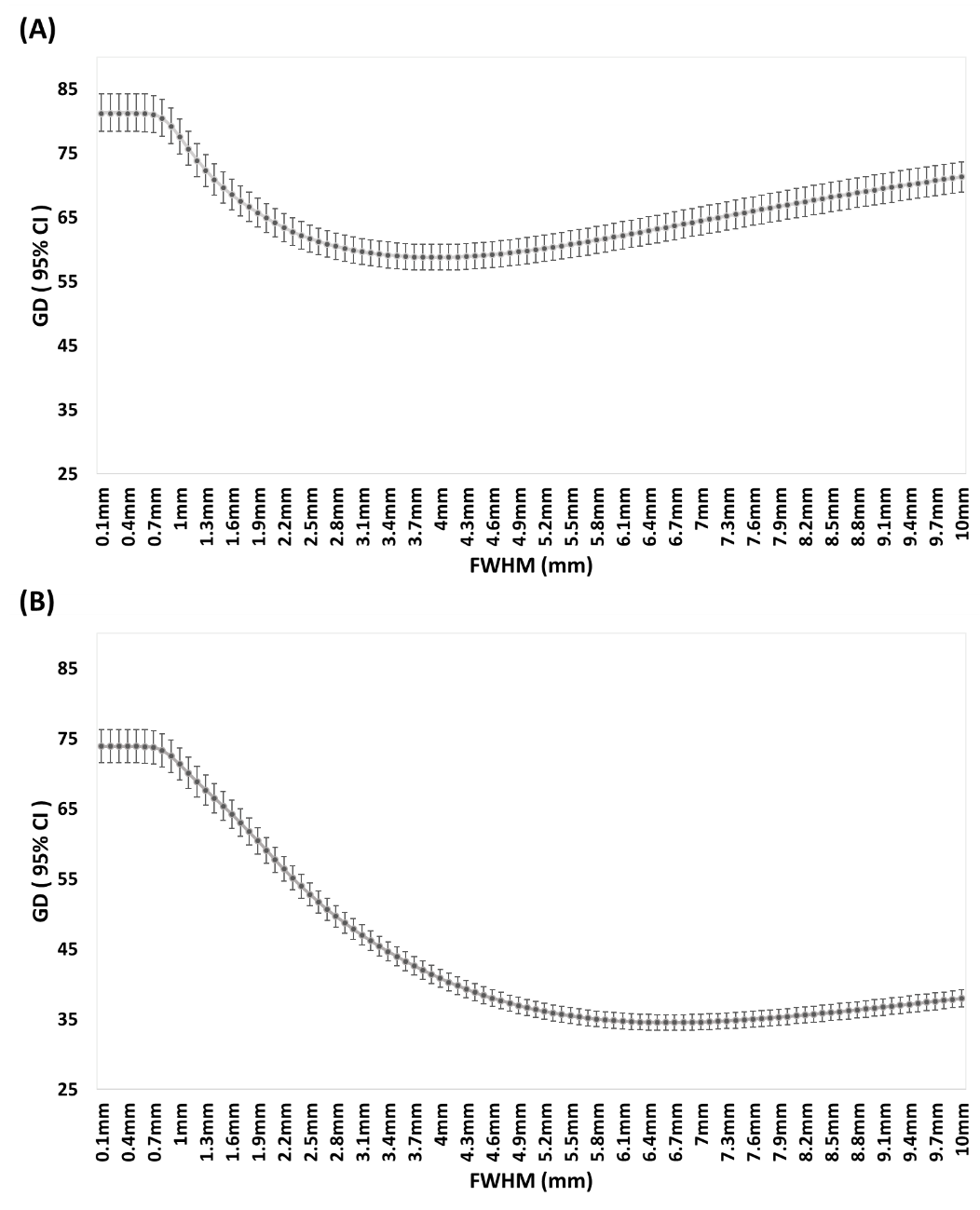


Figure_S4. Mean absolute global difference (±95% confidence interval) between (A) PET and site-specific DRO smoothed for estimating EIR (B) 8 mm smoothed DRO and phantom PET image for estimating harmonization kernel as a function of FWHM of the Gaussian filters. Phantom PET images were acquired for four frames of 5 min. It was reconstructed with ordered subset expectation maximization (OSEM) algorithms, using four iterations and 24 subsets, without any post-smoothing filter.

A subset of PET images (21 scans) with EIR between 4 and 6 mm was selected for evaluating the possibility of harmonizing PET scans with sharper EIR. Figure_S5 (panels A, B, C, D, and E) shows the visual comparison between 5 different scans with EIRs ranging from 4.3 to 5.9mm. A COV% ranging from 6% to 23% is observed in the images between harmonization, and 3 scans have COV%>15%, exceeding the upper acceptable limit of COV% for image quality. Panel A’, B’, C’, and D’ represent PET scans after harmonizing to a target resolution of 6 mm, producing COV% ranging from 5.50% to 9.63%.


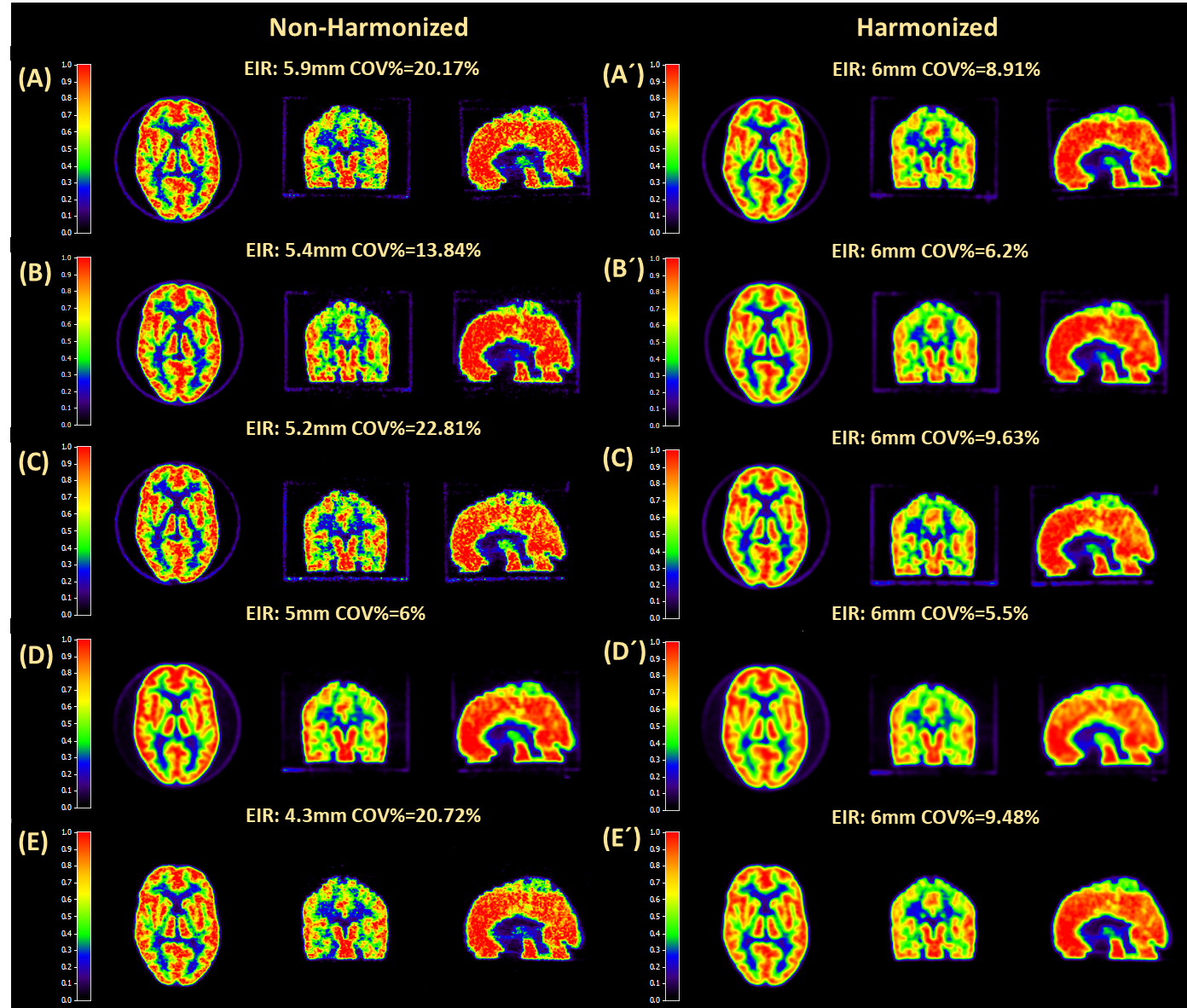


Figure_S5. Visual comparison of phantom PET images acquired in 5 different sites before (A, B, C, D, and E) and after (A´, B´, C´, D´, and E´) harmonization. It should be noted scans with similar EIR can represent different levels of COV% before harmonization, whereas harmonizing images resulted in providing similar image qualities and uniformity without significant signal degradation.

Table_S1 shows image quality and quantitative metrics calculated for scans before and after harmonization. Matching the EIR of the images to 6mm resulted in reducing the variability of COV% from 5.35 to 1.97, and producing images with COV%<15%. However, harmonization did not affect the variabilities of other quantitative metrics (Contrast, GMRC), and all represented similar standard deviations pre and post-harmonization.

| Quantification metrics | Non-Harmonized  Mean±SD(range) | Harmonized  Mean±SD(range) | Difference  Mean±SD(range) |
| --- | --- | --- | --- |
| COV% | **18.21±5.35 (6 to 28.20)** | **8.98±1.97 (5.50 to 12.10)** | **9.22±4.18 (0.50 to 18.54)** |
| GMRC | **0.79±0.01 (0.75 to 0.80)** | **0.75±0.01 (0.72 to 0.78)** | **0.04±0.006 (0.03 to 0.05)** |
| Contrast | **1.85±0.06 (1.66 to 1.92)** | **1.66±0.05 (1.52 to 1.74)** | **0.18±0.03 (0.014 to 0.25)** |
| GMRC_erod_ | **0.97±0.03 (0.92 to 1.04)** | **0.96±0.03 (0.89 to 1.02)** | **0.02±0.002 (0.01 to 0.02)** |
| Contrast_erod_ | **3.61±0.24 (3 to 4.03)** | **3.41±0.23 (2.82 to 3.81)** | **0.20±0.03 (0.15 to 0.30)** |
| Left-to-right GMRC ratio | **1.02±0.008 (0.99 to 1.02)** | **1.02±0.009 (0.99 to 1.03)** | **0.003±0.002 (-0.006 to 0.002)** |
| Cold-spot RC | **0.04±0.01 (0.02 to 0.08)** | **0.05±0.01 (0.03 to 0.08)** | **0.002±0.001 (-0.004 to 0.006)** |

Table_S1. Image quality metrics are measured by toolbox before and after harmonization. This analysis corresponds to 21 scans with EIR ranging from 4 to 5.9 mm, and they were harmonized to result in 6mm EIR. The fourth column represents differences between quantitative metrics before vs after harmonization.

Figure_S6 shows the impact of harmonization on GMRC and contrast for both eroded and non-eroded VOI as a function of COV%. Figure_S6 (A) shows that before harmonization, even though PET scans had similar GMRC, high variabilities were observed in the COV% levels, and only 7 PET scans (33.33%) had COV%<15%. Also, GMRC and contrast of eroded VOI were higher than upper acceptable limits for 14.28% (3 scans) and 8.33% (2 scans) respectively. Left-to-right RC ratio and cold-spot RC were minimally affected by harmonization.


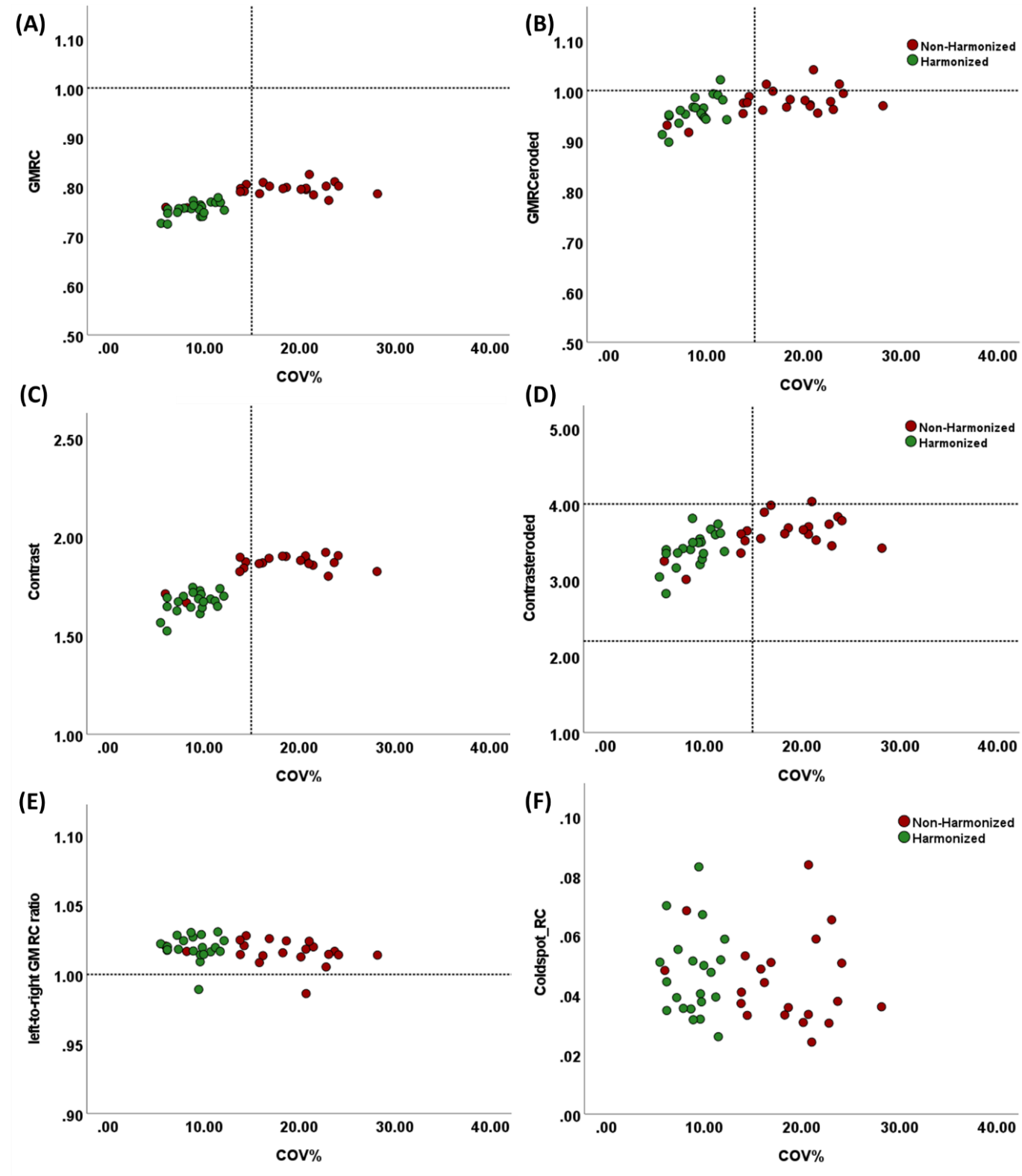


Figure_S6. Comparing GMRC and contrast for (A & C) non-eroded and (B & D) eroded VOIs as a function of COV% for harmonized vs non-harmonized PET images. Panel E and F display left-to-right hemisphere RC ratios and cold-spot RC of different reconstructions for harmonized vs non-harmonized images
